# The prognosis of pain and function in people with hand and thumb base osteoarthritis: a systematic review

**DOI:** 10.1080/07853890.2025.2532113

**Published:** 2025-07-25

**Authors:** Victoria Jansen, Anna Selby, Suzanne Toft, Joanne Greenway, Michelle Marshall, Elaine Nicholls, Danielle A. van der Windt

**Affiliations:** a University Hospitals of Derby and Burton NHS Foundation Trust, Pulvertaft Hand Centre, Kings Treatment Centre, Royal Derby Hospital, Derby, UK; b Centre for Musculoskeletal Health Research, Keele University Faculty of Medicine and Health Sciences, Staffordshire, UK; c Black Country Healthcare NHS Foundation Trust, Knowledge and Library Service, Dorothy Pattison Hospital, Alumwell Close, Walsall, UK

**Keywords:** Disease progression, function, hand, osteoarthritis, pain, symptoms, thumb

## Abstract

**Background:**

This systematic review has summarized evidence regarding the course of hand pain, hand function, and prognostic factors that predict changes in symptoms in people with hand and thumb base osteoarthritis .

**Methods:**

A systematic search of electronic databases was conducted for longitudinal studies on hand osteoarthritis. Two reviewers assessed eligibility quality appraisal, and extracted data on pain, function and prognostic factors. A narrative synthesis was undertaken, and the strength of the evidence was appraised using a modified Grading of Recommendations, Assessment, Development, and Evaluations approach.

**Results:**

Of 9523 articles identified, 24 papers with 8,496 patients met the inclusion criteria. Twelve studies reported on the course of hand pain and 13 on hand function. 25–29% of participants reported worsening pain and 23–59% a deterioration in hand function over 10 years. There is moderate evidence that, on average, function and grip strength deteriorate over time with minimal changes in average hand pain. Twelve studies assessed the prognostic factors for hand OA. Moderate evidence suggests baseline pain and diabetes are associated with changes in hand function and pain;. lower quality evidence for other health and psychological factors.

**Conclusions:**

Over 10 years, there is, little change in hand pain, but some deterioration in hand function. Deterioration in hand pain and function is associated with diabetes and higher baseline pain severity. Further research is very likely to improve our understanding of prognostic factors for symptomatic progression in hand OA.

## KEY MESSAGES

1. Changes over time in hand pain experienced by patients with hand and thumb base OA are, on average, small; grip strength and hand function deteriorate increasingly over time.
2. There is evidence of individual variation in the course of hand pain and function in people with hand and thumb base OA.
3. Perceived hand pain at baseline and diabetes have a moderate level of evidence as prognostic factors that may indicate deteriorating function and pain (respectively), whereas other factors only have low or very low levels of evidence to support them.

## Introduction

Osteoarthritis (OA) is common and frequently affects the hands, with a population prevalence estimate of 42% (radiographic hand OA), which is higher than estimates for the knees (24%) and hips (11%) [1]. Hand OA is often painful and has significant functional and psychological impacts, such as loss of independence or inability to perform a caring role, and can result in early retirement [2]. Hand OA can be divided into different subsets depending on the joints affected: nodal OA, thumb base OA (TBOA), and erosive hand OA [3]. These subsets are described in more detail elsewhere, but essentially the presence of Bouchard and Heberden nodes on the proximal and distal interphalangeal joints of the hand defines nodal OA; thumb base OA is defined as OA in the first carpometacarpal joint and can include the scaphotrapezial trapezoid joint; erosive OA is characterised radiographically by central erosions and collapse of the subchondral bone, and is most commonly seen in the distal interphalangeal joints [4,5].

A previous systematic review on the course of hand OA conducted ten years ago [6] concluded that the literature at that time was predominantly concerned with radiographic progression; further research is needed to understand both the clinical progression of hand OA and the factors associated with poor long-term outcomes. This review intends to focus on TBOA, which is the most common subset of hand OA and frequently occurs in isolation [7]. Patients with TBOA complain of severe pain and difficulty completing everyday essential tasks because of the thumb’s unique role in providing opposition for pinch and dexterity. They feel it is important to know the course of their condition, as they fear losing the use of their hands. Treatment for hand and TBOA includes pharmacological, non-pharmacological and surgical options, and should be tailored to the individual [8]. Non-pharmacological multi-modal treatments are recommended as a first step including education to support self-management, ergonomic task modification, exercises to improve movement, proprioception and strength, and hand splints [8,9].

Understanding the disease course and prognostic indicators is essential for clinicians managing chronic conditions. For example, it has informed models of stratified care and enhanced treatments for those at risk of persistent low back pain [10]. Stratified or personalized care is endorsed by expert working groups as ideal for OA, where the disease course varies [11] and is especially important in conditions with a high prevalence to ensure treatment is targeted at those who need it [12].

Radiographic progression of existing TBOA occurs in 64–71% of patients over 9 years [13]. Visible deformity and significant loss of strength, movement, and hand function are observed in patients with TBOA compared to healthy controls [14,15]. However, weak associations have been reported between the severity of radiographic hand OA and patient-reported measures of pain and function over time [16], indicating that other factors may explain the variation in pain and function outcomes [17]. TBOA is considered more biomechanically driven than finger OA; thus, progression and risk factors may differ [18,19]. Therefore, the aim of this systematic review was to summarize and critically appraise evidence regarding the course and prognosis of symptomatic TBOA, (rather than all subsets of hand OA). Anticipating only a few cohort studies where the prognostic factors for TBOA have been assessed separately from the hand, it is important to consider all research on the course and prognostic factors for hand OA that includes all subtypes (TBOA, nodal and erosive) and, where possible, separate the review evidence that is specific to the thumb base. Therefore, the proposed systematic review considers the following questions.

1. What is the course of pain and functional difficulties in patients with hand OA and TBOA over time?
2. What are the potential prognostic factors associated with pain and function identified in the literature for hand OA and TBOA?

## Methods

The review protocol is registered in the PROSPERO database (CRD42020196725). It has been reported according to the Preferred Reporting Items for Systematic Reviews and Meta-Analyses (PRISMA) guidelines [20].

### Search strategy

The search used keywords and subject-indexed search terms related to the domains of diagnosis (OA), hand, and study design (cohort/prognosis) applicable to each database. The full strategy for the databases searched are presented in Supplementary Tables 1.1 − 1.6.

The following databases were searched on April 13, 2023,: MEDLINE (1946–present), AMED (1995–present), EMBASE (1947–present), PsycINFO (1806–present), CINAHL *via* EBSCO (1937–present), and AgeLine (1978–present). Web-based repositories producing evidence syntheses were also searched using the same keywords in the title or abstract: SIGN, NICE, the TRIP database, and Epistemonikos. Web of Science core databases were searched to identify any further key articles that were not included in the main search. The search was supplemented by a bibliographic screening of relevant systematic reviews identified by the searches.

### Selection criteria

This review included longitudinal studies of adults with a diagnosis of hand OA (including all subtypes) or specifically thumb base OA, where measures of pain and function were collected at a minimum of two time points, at least 12 months apart. Outcomes were examined over the short term (follow-up duration, 12 months to 3 years) and longer term (>3 years). A complete list of the selection criteria is presented in Table 1.

**Table 1.** Inclusion and exclusion criteria.

	Inclusion	Exclusion
Participants	Adult (over 18 years) with a diagnosis of hand OA or primary TBOA confirmed clinically (American College of Rheumatology (ACR) criteria for hand OA, or clinical diagnosis of TBOA and/or radiographic (Kellgren Lawrence ≥2 for hand OA or TBOA or Eaton Littler ≥1 for TBOA).	Traumatic, congenital, or hormonal abnormalities.
*Studies*	Prospective or retrospective observational studies describing the change in hand pain and functional difficulty. Data collection at a minimum of two time points, with follow-up of at least 12-month duration. Any geographic location or healthcare setting.	Assessment of the presence of hand pain or functional difficulty only by (yes/no). Evaluation of a specific intervention for hand OA or TBOA. Published without peer-review or reported only in an abstract. Case studies or qualitative research. Language other than English.
*Outcomes*	Hand pain and hand function. Could also include quality of life, participation, grip, and pinch strength (measured using validated methods).	Radiographic only.

Key: TBOA Thumb base OA.

### Study selection

After the removal of duplicates, titles, abstracts, and full texts were uploaded to Rayyan [21]. Using a pre-specified checklist studies were screened separately by two reviewers (VJ and JG). Any disagreements regarding the inclusion of studies at both stages were discussed and resolved by a third person (MM).

### Data extraction and management

Data were independently extracted by two reviewers (JG and VJ) and verified by a third reviewer (AS) using a bespoke data extraction form. Disagreements were resolved through discussion and consensus among the three reviewers. The data extracted were cohort, country, study setting, population characteristics (age and sex), diagnostic OA criteria used, proportions meeting the criteria, follow-up duration, retention rate, outcome measures (for symptomatic course e.g. hand pain, and function), results, definitions and amount of progression, risk factors, and estimates of their association with pain and function.

### Methodological quality

Methodological quality was assessed by two reviewers (VJ and AS) using the Quality In Prognostic Studies (QUIPS) tool [22]. The QUIPS tool assesses the validity and risk of bias over six domains (participation, attrition, prognostic factor assessment, outcome measurement, confounding factors, statistical analysis, and reporting) to evaluate the overall risk of bias (ROB). Judgements of the overall risk of bias for a study were made as follows: if all domains were classified as low risk, or up to one moderate risk, then this paper was classified as low risk of bias; if one or more domains were classified as high risk, or more than three moderate risk, then this paper was classified as having a high risk of bias [23]. On the prompting items relating to ‘participation and attrition’, cut-offs were used to ensure consistency, with at least 67% for both participation and retention rates considered acceptable [23].

### Evidence synthesis

The following analyses were planned: (1) analysis to pool estimates of change in pain function and any secondary outcomes such as grip strength over time, and to estimate the strength of association between a change in symptoms and individual prognostic factors; (2) An assessment of publication bias using funnel plots to look for bias from ‘small-study effects’; (3) A best evidence narrative synthesis to describe the overall prognosis (outcomes with hand OA) and the value of prognostic indicators for outcome. The latter was conducted using the Grading of Recommendations Assessment, Development, and Evaluation (GRADE) method, modified for prognosis research [24], using the recommended four factors that might lessen confidence in the evidence: risk of bias, inconsistency, indirectness, and imprecision [24–26]. Studies were grouped for synthesis by follow-up length and outcome measured. The study inclusion criteria purposely allowed a broad range of hand OA patients (recruited from a range of settings, with differing diagnostic criteria) to make the results generalizable to all hand and thumb base OA patients, which narrowed our definition of indirectness (Supplementary Table 2). Therefore, indirectness was considered and excluded (as not applicable) as a reason for downgrading the prognostic factor evidence in this review.

## Results

**Figure 1.**
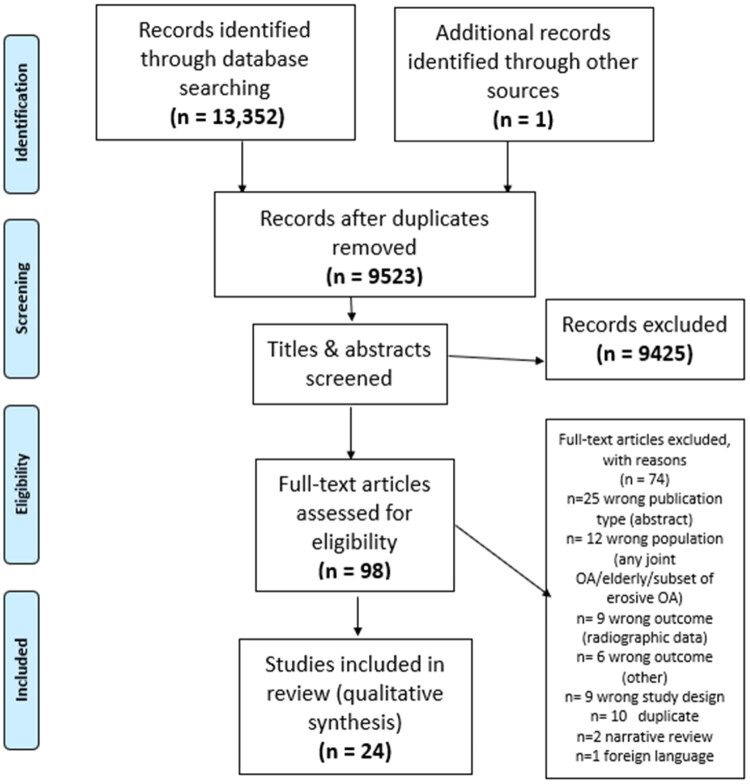
Flowchart of study selection process.

The search strategy identified 13,352 articles. The systematic reviews retrieved were not included in data analysis, but screened for references and one additional study was added. After removal of duplicates 9523 article remained and the titles and abstracts were screened. This resulted in the exclusion of 9425 articles, and 98 were retained for full-text review where a further 74 articles were excluded. Common reasons for exclusion on full-text review were articles published solely as conference abstracts (*n* = 25), studies of OA in which hand data were not presented separately (*n* = 12), cross-sectional studies (*n* = 9), duplicate studies (*n* = 10), and studies providing only baseline outcome data on pain and function (*n* = 6), or follow-up data were exclusively radiographic) (*n* = 9). In total, 24 studies met the selection criteria and were included in the systematic review (Figure 1).

### Study characteristics

The 24 studies included came from 13 different cohorts, and their characteristics are presented in Table 2. Where data from the same cohorts were reported, this was mostly concerned with data from different follow-up time points. Exceptions to this were studies that reported changes in joint-level symptoms versus person-level data for the course of disease or course of disease versus prognostic factor analysis [16,27–29]. The studies were conducted in Europe and the USA with follow-up periods ranged from 1 to 12 years. All studies were prospective observational cohorts, except for one placebo group in a randomized controlled trial of interventions for knee OA [30].

**Table 2.** Characteristics of studies included in the review (*n* = 24).

Study and cohort	Country	Sample size and setting	Age in years (SD/ range)	% Female	Proportions meeting diagnostic criteria for hand OA	Measures of hand pain and hand function
Allen [31] GOGO	USA	*N* = 877 Rheumatology clinics and community	67.7 (8.2)	80%	100% rHOA	AUSCAN (scale not stated), Grip and pinch strength (kg), Global rating of change
Bjisterbosch [39] GARP	Netherlands	*N* = 357 Primary and secondary care, siblings	59.5 (7.4)	83%	100% ACR HOA or rHOA	AUSCAN pain (0–20) Pain on hand joint palpation (0–3) AUSCAN function (0–36)
Courties [30] SEKOIA TRIAL	France	*N* = 869 Part of a RCT treating knee OA	63.8 (7.1)	69%	100% rHOA	AUSCAN pain, function and stiffness (altered scale 0–300) FIHOA (0–30)
Haugen [44] Oslo	Norway	*N* = 128 Rheumatology clinics	67.9 (5.5)	90%	100% clinical diagnosis HOA	AUSCAN pain (0–20) Presence/absence of tenderness on palpation (0–30)
Haugen [16] Oslo	Norway	*N* = 190 Rheumatology clinics	61.5 (5.7)	91%	100% clinical diagnosis HOA	AUSCAN pain (0–20 AUSCAN function (0–36) Presence of pain on palpation (0–20) Grip strength (kg)
Meersseman [32] Belgian HOA Register	Belgium	*N* = 270 Rheumatology clinics, secondary and tertiary care	62.2 (45–84)	86%	100% ACR HOA and rHOA	VAS (0–100) AUSCAN Pain (5–50) ** AUSCAN Function (9–90) FIHOA (0–30) Grip (kg)
McQuillan [33] Stanford	USA	*N* = 92 Secondary care	56.4 (7.6)	53%	100% clinical diagnosis CMCJ OA	AUSCAN pain (0–20) PRWHE pain (0–50) AUSCAN function (0–36) PRWHE Function (0–50)
Damman [28] HOSTAS	Netherlands	*N* = 384 Secondary care rheumatology	60.9 (8.4)	84%	100% ACR HOA	FIHOA (0–30) AUSCAN function (0–36) HAQ for disability (0–3)
Gil [40] Stanford	USA	*N* = 91 Secondary care, hand surgery	NR	NR	100% clinical diagnosis of CMCJ OA	PRWHE pain (0–50) PRWHE function (0–50) AUSCAN pain (0–20) AUSCAN function (0–36)
Van Beest [27] HOSTAS	Netherlands	*N* = 105 Rheumatology clinics	59.2 (7.3)	81%	89% ACR HOA, 100% clinical assessment	AUSCAN pain (0–20) VAS pain (0–100) Pain on palpation (0–8) AUSCAN (function 0–36) Grip (kg)
Botha Scheepers [43] GARP	Netherlands	*N* = 189 Primary and secondary care, and siblings	59.2 (7.3)	79%	75% ACR HOA, 100% clinical diagnosis	AUSCAN pain (0–20) Number of painful hand joints on palpation AUSCAN function (0–36)
Liu [45] HOSTAS	Netherlands	*N* = 314 Secondary care rheumatology	61.4 (8.9)	88%	91% ACR HOA, 100% clinical diagnosis	Pain on palpation (0–90) FIHOA (0–30)
Magnusson [29] Oslo	Norway	*N* = 209 Secondary care rheumatology	61.6 (5.6)	91%	80% ACR HOA, 100% clinical diagnosis	AUSCAN pain (0–20) Number of joints with symptomatic HOA AUSCAN function (0–36)
Vanhaverbeke [46] Belgian HOA Register	Belgium	*N* = 270 Rheumatology clinics, secondary and tertiary care	62.9 (8.0)	85%	100% ACR HOA and rHOA.	VAS pain (0–100) AUSCAN pain (0–50) Number of tender hand joints VAS function (0–100) AUSCAN function (0–90) FIHOA (0–30)
Neuprez [38] LIHOC	Belgium	*N* = 203 Specialist MSK clinic tertiary care.	69 (61–75) Median (IQR)	90%	100% rHOA and clinical HOA	VAS pain (0–100) AUSCAN pain (0–100) AUSCAN stiffness (0–100) AUSCAN function (0–100) Number of painful hand joints Number of tender hand joints on pressure Number of hand soft tissue swellings
Snyder [48] JoCoOA	USA	*N* = 327 Community	63.1 (7.4)	72%	100% rHOA and clinical HOA	Number of symptomatic hand joints Number of tender hand joints on palpation (0–30)
Studies below also included participants without a diagnosis of hand OA						
Dieppe [34] Bristol 500	UK	*N* = 500 Rheumatology clinic	59 (9.4) hand alone, 64 (9.3) hand and knee	86%	53% rHOA and symptomatic HOA	Self-report hand pain intensity (none/mild/moderate severe) Reported change in index joint (better/same/ worse) Reported change in overall hand condition (better/ same/worse) since baseline. Reported change in index joint (better/same/ worse)
Cvijetic [35]	Croatia	*N* = 286 Rural community	54.9 (9.5) males 56.4 (8.4) females	56%	rHOA 36% males, 32% females.	Grip strength (kilopounds)
Ding [36]	Finland	*N* = 543 Finnish dental and teacher’s associations	54.5	100%	19% rHOA	Joint pain hand diagram (no pain = 0/mild = 1/moderate = 2/ severe= 3)
Marshall [41] CASHA	UK	*N* = 432 General population	64.2 (8.2)	62%	30% ACR HOA, 76% rHOA in 1jt	AUSCAN pain (0–20) AUSCAN function (0–36) Global perceived change
Marshall [7] CASHA and CASK	UK	*N* = 1167 General population	64.8 (8.3)	60%	65% rHOA and symptomatic HOA, 35% unclassified as symptomatic with KL ≤ 2	AUSCAN pain (0–20) AUSCAN function (0–36)
Scherzer [42] JoCoOA and GOGO	USA	*N* = 872 Rheumatology and community	59.5 (7.4)	68%	8% rHOA GOGO criteria*	AUSCAN pain (0–20) AUSCAN function (0–36)
Siviero [37] EPOSA	Italy/Europe	*N* = 2942 Population-based study involving cohorts of adults between the ages of 65 and 85 residing in Germany, Italy, Netherlands, Spain, Sweden and UK	73.7 (5.0)	52%	17% clinical HOA	AUSCAN pain (0–20) AUSCAN function (0–36) Grip strength (kg)
Van Beest [49] HOSTAS	Netherlands	*N* = 202 Secondary care rheumatology	60.7 (8.3)	83%	92% ACR HOA	VAS right hand pain (0–100) AUSCAN pain (0–20) AUSCAN function (0–36) Thumb pain and stiffness: present/absent Presence of pain on palpation right thumb base (0–3) Presence of bony swelling on palpation right thumb base (absent/present)

Key: rHOA radiographic hand OA, GROC, global rating of change, AUSCAN Australian Canadian Hand OA index, FIHOA Functional Index for hand OA, PRWHE patient rated wrist and hand evaluation, VAS visual analogue score, RCT randomised controlled trial, HAQ Health Assessment Questionnaire, MCII minimum clinically important improvement, CMCJ carpometacarpal, MSK musculoskeletal, IQR interquartile range.

VAS, AUSCAN, FIHOA and PRWHE greater score = worse symptoms.

*GOGO criteria for rHOA: KL ≥ 2 in ≥3 joints across both hands with 2 joints in the same group (DIP/PIP/CMC). ACR HOA American College of Rheumatology Criteria for hand OA: pain, aching or stiffness in the hand and 3 of the following – hard tissue enlargement of at 2 or more of the following joints (2nd/ 3rd distal interphalangeal joints/proximal interphalangeal joints/ first carpometacarpal joints); hard tissue enlargement of 2 or more distal interphalangeal joints, deformity of at least one of the listed joints; less than 3 swollen metacarpophalangeal joints.

**Scoring for AUSCAN unusual with a lowest score of 5 not 0.

### Participants

The total number of participants included in the 13 cohorts was *n* = 8,699 [7,^28–39^], with sample sizes ranging between 91 [40] and 2942 [40] (Table 2). The average age of the samples ranged from 54.5 to 73.7 years [36,37], with predominantly female populations of 52 to 100% [36,37]. Participants were recruited from the community [7,31,35,37,41,42], from those attending primary [39,43], secondary [16,27–29,31,33,40,44,45] and tertiary [32,46] healthcare settings, and in one study from teachers’ and dentists’ professional associations [36]. Four cohorts used clinical criteria to determine a diagnosis using the American College of Rheumatology (ACR) [47] or clinician-diagnosed hand OA [28,29,33,37]. Four studies used specified radiographic criteria alone [30,31,35,36], and five used a combination of the ACR criteria or specified clinical signs and radiographic criteria [7,34,38,39,46].

When considering the different subgroups of hand OA, most cohorts did not report any data on the different subgroups of hand OA, three studies provided data on the proportion of a subtype in the study population [38,39,48] and one study provided outcome data separated by the different subtypes [7]. One cohort included exclusively people with TBOA [33,40], one study presented only the data for those with TBOA who had a magnetic resonance imaging (MRI) scan [49] and two hand OA studies presented separate data for those with TBOA [7,39].

### Methodological quality

Five studies (24%) had an overall low risk of bias [7,27,28,37,39] the majority (*n* = 15, 71%) were rated as having an overall high risk of bias (Table 3). The domain most considered to have a high risk of bias was the confounding factor domain (*n* = 11), where confounding factors were not measured [33,35,40], well described [29,30,34,36,41,43,44,46] or accounted for in the study design or analysis [36,43]. Four studies without any domains scored as having a high risk of bias had a moderate risk of bias in three domains, giving them an overall high risk of bias [31,38,45,49].

**Table 3.** Risk of bias of studies included in the review (*n* = 24).

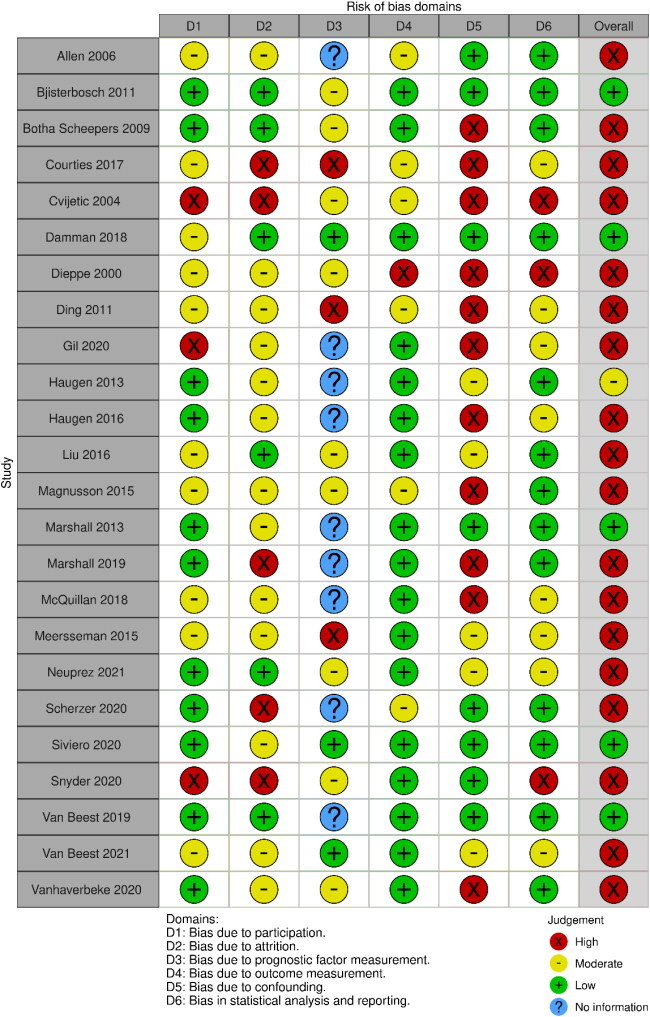
QUIPS Quality in prognosis studies tool D3 bias due to prognostic factor measurement: No information present as these studies did not assess prognostic factors.

### Publication bias

Publications were assessed to confirm that the data presented were complete, as described in the methods section of the publication. The number of studies reporting the same outcome or assessing the same prognostic factor was too small to allow a more formal analysis of reporting bias using funnel plots [50].

### Measures of hand pain and function

The Australian Canadian Hand OA Index (AUSCAN) was the most commonly used measure to assess pain and function [51]. The AUSCAN pain and function subscales were reported in 15 studies [7,16,27,29,32,33,37–43,46,49] (Table 2). The severity of pain on palpation of individual joints was reported in six studies [16,27,39,44,45,49]. Pain intensity was assessed using the Visual analogue scale VAS (0–100 mm) in five studies [27,32,38,46,49]. Patient-rated wrist and hand evaluation (PRWHE) for pain and function [52] was used in two studies from the same cohort [33,40]. The functional Index for Hand OA (FIHOA) [53] was collected in five studies [28,30,32,45,46]. Grip strength was measured in six studies [16,27,31,32,35,37] using various definitions and methods of reporting results. Pinch strength was reported in only one study [31].

The data presented for all measures of hand pain and function varied widely. For example, with patient-rated outcome measures, some studies used total scores, others subscale scores, some presented change scores rather than follow-up scores, and some categorized data such as those who had improved, remained stable, or deteriorated. For those studies that grouped participants into categories of improving, stable, or worsening, the way in which this was done also varied as follows: a patient global rating of change question [34,41]; a change in the number of symptomatic joints [29]; the change from baseline regardless of magnitude [30]; a change from baseline score that exceeded the minimum clinically important difference for the patient-rated outcome measure [16,28,37,39,46]; and grouping patients as low or high pain or function and describing the proportions who transitioned between groups during follow up [42].

### Clinical course of hand pain

Twelve studies reported the course of hand pain at the person level over time [7,16,29,32–34,38–40,43,44,46] (Table 4). The data were unsuitable for meta-analysis owing to variability in the outcome measures used, the way data were presented, length of follow-up, and heterogeneity in terms of study setting and participants. When considering the course of hand OA pain, six studies showed average reductions in hand pain [32,33,38,40,44,46] and an equal number of average increases in hand pain [7,16,29,34,39,43]. The average change over time reported in most studies was small, despite the time frame examined [32,44,46]. For example, the mean deterioration in the AUSCAN pain subscale score was reported as 1.0 (SD 4.0, 95% CI 0.4 − 1.6) on a 0 to 20 scale over 3 years [43] and another study reported 0.7 (95% CI 0.3–1.2) [39] over four to seven years (Table 4). Two studies presented the proportions of participants with improvements or deterioration in pain scores, over two years 25% had deteriorated [38], over seven years 29% had deteriorated and 21% had improved [16]. The global rating of change for hand pain and stiffness was assessed, with 49% reporting worse symptoms after four years [31]. One study [41] assessed changes in ‘hand problem’ rather than hand pain and found that 56% of participants had a worsening problem over seven years. Another study [34] assessed the change in hand pain with a change in the overall condition, finding deterioration in similar proportions at three (48%) and eight years (54%).

**Table 4.** Study outcomes.

**Study: author year** and **Cohort.**	**Follow up: years (SD)** and **attrition (%) retained sample (*n*)**	**Outcomes** mean (SD) unless otherwise stated			**Deterioration in hand pain** and **function**	
		Baseline	Follow up	**Outcome change** Mean (SD) unless otherwise stated	Y/N	Detail
**Setting: Rheumatology/secondary care clinics** **Participants: 100% HOA**						
Allen [31] GOGO	4.1 (1.1) 39% *n* = 531	Hand pain and function			Y Deterioration hand pain and function	Small average deterioration pain and function (may not be clinically significant). 43–49% rated as globally had worsened. No: Grip strength did not deteriorate overall, pinch strength reduced.
		AUSCAN 34.0 (12.3)	35.3 (13.0)	AUSCAN 1.3 (10.4)		
		Grip and pinch strength (Kg)				
		L: 46.9 (24.0) R: 50 (23.6)	L: 47.5 (20.6) R: 50 (20.5)	R: −0.4 (15.4) L: 0.4 (16.3)		
		L: 12.0 (6.1) R: 12.3 (5.9)	L: 11.4 (4.7) R: 12.0 (5.0)	R: −0.4 (6.3) L: −0.7 (7.0)		
Haugen [44] Oslo	4.7 (0.4) 32% *N* = 87	Hand pain			N Improved hand pain	Improvements in pain (likely clinically significant as median is 20% change, MCII used elsewhere is 1.4)
		NR	NR	AUSCAN Median change (IQR) −4.0 (–6.0, −2.0)		
Haugen [16] Oslo	7 41% *n* = 112			Hand pain	Y Deterioration hand pain and function	Variable: average small deterioration in pain, function, and strength, clinically significant in 47%, but 32% improved.
				AUSCAN 0.8 (3.4) Improvements beyond MCII pain 21.1%, worsening beyond MCII pain 28.9%		
				Hand function		
				AUSCAN 1.2 (6.3) Improvements beyond MCII 31.6%, worsening 46.9%		
				Grip strength		
				R −0.7 (6.9), L: −1.1 (6.9)		
				Variable: pain and number of tender joints reduced. Function and strength deteriorated. Authors felt disease progressed more rapidly early on, focus on erosive OA.		
Meersseman [32] Belgian HOA Register	5.8 43% *n* = 154	Hand pain			N Improved hand pain Y Deterioration hand function	Variable: pain and number of tender joints reduced. Function and strength deteriorated. Authors felt disease progressed more rapidly early on, focus on erosive OA.
		VAS 41.6 (25.0) AUSCAN 20.5 (10.5)	33.3 (28.2) 17.2 (10.8)			
		Hand function				
		FIHOA 8.7 (6.4) AUSCAN 39.5 (21.0)	9.8 (6.5) 40.2 (20.0)			
		Grip strength (kg)				
		Dominant 19.1 (8.6) Nondominant 18.5 (8.1)	15.9 (8.3) 15.0 (7.8)			
		No. painful joints				
		3.4 (3.3)	2.8 (3.4)			
		No. tender hand joints				
		3.0 (3.1)	4.8 (3. 8)	Median (IQR) −2.0 (–5.0 to 1.0)		
Damman [28] HOSTAS	2 19% *n* = 312	NR	NR	Hand function proportions given are those with worsening function/ total followed up	Y Deterioration hand function	Deteriorating function, BUT better understanding and less emotions and consequences perceived because of their OA. No: 50% did not deteriorate in function.
				FIHOA 3.7 (2.6), 50% (157/311) AUSCAN 5.6 (3.6), 37% (117/314 change > MCII)		
				Disability		
				HAQ 0.4 (0.2)		
Van Beest [27] HOSTAS	2 19% *n* = 85	NR	NR	Pain: Joint level changes	N Improved hand pain	65% of joints with pain at baseline had reduced pain Yes: 16% of joints had increased pain. A decrease in synovitis (not BML) was associated with less pain.
				65% (76/116) of painful joints at baseline had less pain at FU. 16% (115/678) joints that has pain below the maximum score increased pain at FU.		
Liu [45] HOSTAS	1 55% *n* = 173	Hand pain NR	NR	NR	Y Deterioration hand function	Function deteriorated (over half the sample is missing)
		Hand function				
		FIHOA 8.0 (range 0–24)	9.0 (0–28)			
McQuillan [33] Stanford	1.5 10% *n* = 83	Hand pain		NR	N Improved hand pain and function	Pain reduced, and function improved. Early-stage radiographic disease, surgical treatments provided, and those cases excluded from FU.
		AUSCAN (+ range) 6.6 (4.1; 0–18) PRWHE (+ range) 17.4 (11.7; 0–44)	6.4 (4.0; 0–19) (19.7; 0–19) 15.8 (10.6; 0–48)			
		Hand function		NR		
		AUSCAN (+ range) 10.9 (7.7; 0–31) PRWHE (+ range) 11.3 (10.8; 0–44.5)	10.3 (6.5; 0–33) 9.8 (9.7; 0–46)			
Gil [40] Stanford.	3 7% *n* = 85	Hand pain		NR	N Improved hand pain and function	Pain and function improved. Early radiographic stage of disease.
		PRWHE 17.2 (11.5) AUSCAN 6.6 (4.1)	18mo 15.6 (10.6) 36mo 16.0 (11.3) 18mo 6.1 (4.0) 36mo 6.4 (4.2)			
		Hand function		NR		
		PRWHE 13.0 (13.4) AUSCAN 10.8 (7.7)	18mo 10.8 (12.1) 36mo 11.1 (12.3) 18mo 10.8 (12.1) 36mo 11.1 (12.3)			
Magnusson [29] Oslo	7 51% *n* = 103	NR	NR	Hand pain	Y Deterioration hand pain and function	Average changes not clinically significant
				AUSCAN 0.7 (3.6)		
				Hand function		
				AUSCAN 1.1 (6.4)		
				Change in no. Symptomatic hand joints		
				0.9 (3.1)		
Vanhaverbeke [46] Belgian HOA Register	9.7 61% *n* = 106	Hand pain			N Hand pain improved. Y Hand function deteriorated	Pain and number of tender joints reduced, MCII for AUSCAN. Small deterioration in function 59% and significant deterioration in grip strength (over half the sample was missing but long FU).
		AUSCAN 19.6 (10.9) VAS 40.2 (25.1)	T1: 17.2 (10.8) T2: 17.4 (10.6) (*p* = 0.073) T1: 33.3 (28.2) T2: 38.6 (27.2 (*p* = 0.656)	Over 10 years mean differences AUSCAN −1.84 (NS) VAS −1.16 (NS)		
		Hand function				
		AUSCAN T0: 39.3 (21.3) FIHOA T0: 8.8 (6.5)	T1: 40.2 (20.0) T2: 41.9 (22.0) *p* = 0.035* T1: 9.8 (6.5) T2: 9.9 (6.8) *p* = 0.017*	4.52 ± 19.91 59% (participants) showed functional deterioration. FIHOA 1.45		
		Grip strength (kg)				
		T0: 19.0 (9.0)	T1: 15.9 (8.3) T2: 15.8 (8.0) *p* < 0.001*	Dominant hand (*p* < 0.001)		
		No. Swollen hand joints				
		T0: 1.4 (1.8)	T1:2.6 (2.7) T2:3.6 (2.9)	Nos swollen joints increased (*p* < 0.001)		
		No. tender hand joints				
		T0: 3.1 (3.4)	T1: 4.8 (3.8) T2:2.3 (3.5)	No. tender joints decreased (*p* = 0.035)		
Neuprez [38] LIHOC	2, 13% *n* = 176	Hand pain			N No significant changes	Average changes not significant. Radiological progression was not associated with changes in self-reported pain and function.
		VAS 44.2 (23.7) AUSCAN 44.3(27.4)	42.9 (27.7) 41.5(27.3).	25% deterioration >8 MCID AUSCAN pain		
		Hand function				
		AUSCAN 45.9 (27.0)	48 (28.2)	34.7% deterioration >15 MCID total AUSCAN score		
		No. painful hand joints				
		3.57 (6.2)	3.34 (5.7)			
		No. tender hand joints				
		7.9 (8.6)	7.8 (8.6)			
**Setting: Includes primary care** and **community** **Participants: 100% HOA**						
Bjisterbosch [39], GARP	6.1 (range 5–7.8) 19% *n* = 289	Hand pain			Y Deterioration hand pain and function	Yes: small average deterioration (may not be clinically significant)
		AUSCAN 6.7 (4.8)	7.4(4.9)	0.7 95% CI (0.3–1.2)		
		Hand function				
		AUSCAN 11.8 (8.9)	13.9 (8.9)	2.1 95% CI (1.3–2.9)		
		Hand Joint palpation				
		NR	NR	NR		
Botha Scheepers [43], GARP	2, 9% *n* = 172	Hand pain			Y Deterioration hand pain and function	Yes: small deterioration in pain and function may not be clinically significant.
		AUSCAN 6.2 (4.6)	7.2 (4.8)	1.0 (4.0), 95% CI 0.4 to 1.6, SRM 0.25		
		Hand function				
		AUSCAN 11.1 (8.8)	12.5 (9.1)	AUSCAN 1.4 (6.1) 95% CI 0.5 to 2.3, SRM 0.23		
Snyder [48], Jo Co OA	12, 0% *n* = 327	Hand pain			N Hand pain	No: Majority did not experience symptoms in additional joints. No measures of severity of pain.
		NR	NR	31% experienced symptoms in additional joint(s) over 12 years. Change in no. symptomatic joints was median (IQR) 0(0–1).		
Courties [30]	2.6 (0.7) *n* = 307 (65%)	Hand pain			N Hand function	Small average improvement FIHOA (may not be clinically significant – over half the sample are missing). rHOA not symptomatic.
		Reports AUSCAN score worsened (no follow-up data presented)		NR		
		Hand function				
		FIHOA 3.9 (4.8)	3.2 (4.4)	23% (*n* = 38/164) showed deterioration in FIHOA score		
**Setting: Rheumatology/ Secondary Care** **Participants: Not 100% HOA**						
Dieppe [34]	8 30% *n* = 349	Hand pain and change in hand pain and overall condition.			Y Deterioration in hand condition	Proportions with severe pain have increased and those with no pain have reduced at both time points. Majority describe deterioration at 8 years. With HOA alone 54% experienced progression, with hand and knee OA 71% – in the index joint. At each time point some improved suggests a variable course
		Hand only: none 10%, mild 41%, moderate 39%, severe 10%	3 years: none 5%, mild 40%, moderate 46%, severe 10% 8 years: none 8%, mild 36%, moderate 38%, severe 18%	Self-reported change index joint hand OA only: 3 years: better 23%, same 28%, worse 48% 8 years: better 27%, same 20%, worse 54%		
		Hand and knee OA: none 3%, mild 23%, moderate 38%, severe 35%	3year: none 2%, mild 22%, moderate 46%, severe 29% 8 years: none 2%, mild 10%, moderate 50%, severe 37%	Self-reported change index joint hand and knee OA: 3 years: better 16%, same 16%, worse 68% 8 years: better 14%, same 15%, worse 71%		
Van Beest [49] HOSTAS	2 18% *n* = 165	Hand pain			N Majority remained stable	Reports on thumb base pain only, not hand pain or hand function at 2 yrs. MRI and radiographic scores also remained stable in the majority.
		NR	NR	Self-report thumb base: pain decreased 26/165 joints = 16%, pain stable 121/165 = 73%, worsening 18/165 = 11%. stiffness decreased 23/165 = 14%, stable 124/165 = 75%, worse 18/165 = 11%. Pain on palpation decreased/resolved in 32 patients (19.4%), increased or developed in 33 patients 20%.		
**Setting includes primary care** and **community.** **Participants: not 100% HOA**						
Cvijetic [35]	10 0% *n* = 286	Strength Grip (Kp)			Y Deterioration in grip strength	Over 10 years males lost an average of 5 kp/ 7% and females an average of 1.5–3 Kp or 4–9% of their grip strength.
		Males R 69.8 (22.1) L 68.6 (24.7)	R 65 (24.3) L 63.5 (20.9)	NR		
		Females R 35.8 (13.6) L 31.9 (16.5)	R 32.7 (13.2) L 30.5 (12.9)			
Ding [36]	5 12% *n* = 482	Hand pain			Unclear	High probability of persistent pain but severity not described (some may have worsened some stayed the same). 8.3% improved right hand and 12% left.
		8.3% had pain at baseline (severity not reported)		*Results only pertain to those symptomatic at baseline the probability of persistent pain in the radial digits for dentists was 0.93 and 0.79 in teachers.		
Marshall [41] CASHA	7 33% *n* = 253	Hand pain		Global perceived change in hand problem	Y Deterioration in hand problem	Variable but majority (56%) deteriorated.
		AUSCAN baseline scores not presented only scores within photographic change groups so unusable		improved: 17%, no change: 26%, deteriorated: 56%		
Marshall [7] CASHA/CASK	3 10% *n* = 963	Hand pain			Y Deterioration in pain and function	Yes: with OA meeting subgroup criteria No: for those not meeting subgroup criteria, and erosive OA higher pain levels but no deterioration.
		AUSCAN (and 95% CI) Thumb and IPJ: 6.3 (5.4,7.2) Thumb: 6.6 (6.0, 7.2) Hand: 6.8 (6.2, 7.5) Erosive: 8.0 (6.7, 9.2)	AUSCAN (and 95% CI) Thumb and IPJ: 8 (7.1,8.8) Thumb: 7 .1 (6.6, 7.7) Hand: 7.7 (7.0,8.3) Erosive: 8.0 (6.8, 9.2)	Unclassified group little or no change in pain in 3 years, all other groups showed small increases pain (0.5–1.5)		
		Hand function				
		AUSCAN IPJ and thumb: 9.9 (8.1, 11.7) Thumb: 10.6 (9.5, 11.8) Hand OA: 11.1 (9.8, 12.5) Erosive: 13.5 (11.0, 15.9)	AUSCAN IPJ and thumb: 12.6 (11.2, 13.9) Thumb: 11.3 (10.5, 12.2) Hand OA: 11.9 (10.9, 12.9) Erosive: 13.9 (12.1, 15.6).	Unclassified group little or no change in disability in 3 years		
Scherzer [42] JoCoOA/GOGO	12 3% *n* = 845	Hand pain			N Majority unchanged pain	Variable small numbers in the change groups, majority not in high or low pain groups and unchanged (all numbers increased included incident hand OA over follow up)
		GOGO low pain AUSCAN: 37 (4.4%) GOGO high pain AUSCAN:27 (3.2%)	6yrs 85 (10.4%) 12yrs 112 (13.3%) 6yrs 49 (6%) 12yrs 88 (10.4%)	Low pain to high pain 36 High to low pain 20		
Siviero [37] EPOSA	1.5 36% *n* = 1842	Hand pain			N Majority unchanged	Unclear – only 16.7% met ACR criteria for hand OA (the average AUSCAN hand pain score indicates hand problems).
		NR – reports weighted baseline AUSCAN pain whole group 7.6 (15.2) Baseline scores for 2 groups: worse 10 (16.6) and not worse 6.6 (14.7), but no FU scores. *n* = 453, not worse *n* = 1,389).		NR		
		Hand function				
		AUSCAN worsening group 10.4 (±14.73) not worse 6.6 (14.7) 8.7 (±15.6)	24.5 (±18.1),	MCID score of AUSCAN function +4 ‘worse’ identified 453 (24.4%) with physical function decline at 12–18 months. MCID −2 ‘improved’ identified 23.5% (432) (total *n* = 1842, worse *n* = 453, not worse *n* = 1,389)		

Key: rHOA radiographic hand OA, GROC, global rating of change, AUSCAN Australian Canadian Hand OA index, FIHOA Functional Index for hand OA, PRWHE patient rated wrist and hand evaluation, VAS visual analogue score, HAQ Health Assessment Questionnaire RCT randomised controlled trial, MCII minimum clinically important improvement, CMCJ carpometacarpal, MSK musculoskeletal, IQR interquartile range.

VAS, AUSCAN, FIHOA and PRWHE greater score = worse symptoms.

SD standard deviation, IQR interquartile range, SRM standardised response mean, NR not reported, NS not statistically significant, MCII minimum clinically important improvement, CMCJ carpometacarpal, No. number of, mo months, yr year, L left, R right, FU Follow up.

*GOGO criteria for rHOA: KL ≥ 2 in ≥ 3 joints across both hands with 2 joints in the same group (DIP/PIP/CMC). ACR HOA American College of Rheumatology Criteria for hand OA: pain, aching or stiffness in the hand and 3 of the following – hard tissue enlargement of at 2 or more of the following joints (2nd/ 3rd distal interphalangeal joints/proximal interphalangeal joints/ first carpometacarpal joints); hard tissue enlargement of 2 or more distal interphalangeal joints, deformity of at least one of the listed joints; less than 3 swollen metacarpophalangeal joints.

Only four studies assessed thumb base OA as a separate phenotype. Two studies from the same cohort of clinically diagnosed early thumb base OA suggested that, on average, hand pain was reduced by small amounts. AUSCAN Scores on 0–20 scale were reduced from 6.6 (4.1) to 6.4 (4.0) over both 18 months [33] and three years [40] with large standard deviations and a wide range in pain scores (Table 4). Another study with small mean changes in pain in hand OA stated that over six years pain scores remained above the patient’s acceptable symptom state in 54.4% of those with clinical and radiographic thumb base OA [39]. A final study reported on the presence or absence of pain in thumb base OA over two years, pain decreased in 16%, remained stable in the majority (73%), and worsened in 11% [49].

Five studies reported on the course of hand pain at the joint level. A study found that 65% of the joints with pain at baseline had reduced pain at two years and 16% of the joints had increased pain [27]. Over eight years 23% reported improvement, and 54% had worsened pain in an index hand joint [29,46]. Two studies found that on average over seven to ten years the severity of pain did not change [29,46] and the number of painful joints reduced from an average of 3.1 (SD 3.4) to 2.3 (SD 3.5) (*p* = 0.035) but the number of swollen joints increased from an average of 1.4 (SD 1.8) to 3.6 (SD 2.9) (*p* < 0.001) [46]. A third study found that 31% of participants experienced symptoms in additional joints (s) over 12 years, with little change in the overall number of symptomatic joints (median, IQR) 0(0–1) [48].

The GRADE assessment of 11 prospective studies with 3051 participants found low and very low-level evidence for an average change in hand pain over both the short and long term (Table 5). The evidence was downgraded as most of the studies had a high risk of bias, inconsistency between studies for the direction of mean pain changes, the short-term studies also had small sample sizes, and the longer-duration studies had different approaches to reporting pain scores.

**Table 5.** GRADE assessment: summary of findings on prognosis by outcome.

			1	2	3	4	
Outcome (follow-up time)	Evidence base (no. studies; relevant sample size)	Range of effect	Quality of studies for this outcome	Inconsistency	Indirectness	Imprecision	Strength of evidence (amended GRADE)
Change in hand pain (1–3 years)	6 prospective studies [7,^27^,^33^,^38^,^40^,^43^]. *n* = 1847	At person level average AUSCAN pain changed very little increased 0.5–1.5 (0–20 scale) or decreased. Estimate for proportion with worsening pain are 25%.	−1	−1	+1	−1	⊕□□□ very low
Change in hand pain (4–10 years)	5 prospective studies [16,29,32,39,46]. *n* = 1296	At person level average AUSCAN pain changed very little increased 0.7–0.8 (0–20 scale) or decreased. Estimate for proportion with worsening pain 29%.	−1	−1	+1	+1	⊕⊕□□ low
Change in hand function (1–3 years)	9 prospective studies^,^ [7,^28^,^30^,^33^,^37^,^38^,^40^,^43^,^45^].^^ *n* = 6179	At person level change scores for function deteriorated from an average of 1.4–5.6 on AUSCAN (0–36 scale). Estimates for proportion with worsening function were 23–42%.	−1	−1	+1	+1	⊕⊕□□ low
Change in hand function (4–10 years)	5 prospective studies [16,29,32,39,46] *n* = 1296	Small average deterioration in function, average change score between 1.1 and 2.1 (0–36 scale) Estimates for proportion with worsening function were 47–59%.	−1	+1	+1	+1	⊕⊕⊕□ moderate
Changes in grip or pinch strength (4–10 years)	5 prospective studies [16,31,32,35,46]; *n* = 1893	Small mean changes in grip short- term (–1.1– +0.4 kg) with a trend for greater deterioration over longer time periods.	−1	+1	+1	+1	⊕⊕⊕□ moderate

Key: AUSCAN Australian Canadian Hand OA index.

**Conceptualization: Quality of evidence across studies**.

⊕⊕⊕⊕ High = Further research is very unlikely to change our confidence in the estimate of effect.

⊕⊕⊕□ Moderate = Further research is likely to have an important impact on our confidence in the estimate of effect and may change the estimate.

⊕⊕□□ Low = Further research is very likely to have an important impact on our confidence in the estimate of effect and is likely to change the estimate.

⊕□□□ Very low = Any estimate of effect is very uncertain.

### Clinical course of function in hand OA

Thirteen studies reported changes in function at the person-level over time [7,16,28–30,32,33,37–40,43–46], with variations in the magnitude of change (Table 4). Heterogeneity between studies precluded meta-analysis of the results. Over the short term (up to three years), three studies showed average improvements in function [30,33,40], and five deterioration [7,28,38,43,45]. The reported magnitude of the change in the short term varied from small mean change scores [7,43] for example, AUSCAN function mean change on a 0–36 scale 1.4 (6.1) [7,43] to larger mean changes of 5.6 (3.6) and for FIHOA mean change on a 0–30 scale of 3.7 (2.6) [28]; (increased scores represent a deterioration). Estimates from four studies for proportions of participants with deterioration in function in less than three years ranged from 23–42% [28,30,37,38], with functional improvement in 24% [37]. Over four to ten years of follow-up, the estimates for the proportions with deterioration in function were between 47 and 59% [16,46], and improvements were seen in 32% [16] of the participants.

The GRADE assessment found low-level evidence of small changes in hand function in the short term. This included nine prospective studies with 6179 participants, the level of evidence was downgraded due to the number of studies at high risk of bias and inconsistency due to different: directions of change; outcome measures; and reporting of outcome data. Moderate-level evidence for small changes in hand function over long-term follow-up was found in five studies with 1296 participants. Three studies had a high risk of bias, leading to downgrading in one domain. These studies demonstrated an average worsening function score in most patients with hand OA (Table 5).

Baseline and follow-up grip strength scores were reported in five studies [16,31,32,35,46] (Table 4). These demonstrated that small changes in grip strength occurred, in one study over four years mean change grip right −0.4 kg (SD 15.4), left 0.4 kg (SD 16.3) [31], with greater reductions in grip strength over time (at seven years mean change −0.7 kg (SD 6.9) in right, and −1.1 kg in the left hand (SD 6.9) [16] and over ten years mean change from a baseline score of 19.0 kg (SD 9.0) to 15.8 kg (SD 8.0)) [32] at follow-up.

On GRADE assessment of the five prospective studies with 1893 participants, a moderate level of evidence was found for small mean reductions in grip strength with a trend for greater deterioration over longer time periods (Table 5). Three studies had a high risk of bias, which led to a downgrading in a single domain.

### Candidate prognostic factors

Twelve studies assessed 46 potential prognostic factors, which were grouped under the following six headings: demographic and social characteristics, symptom severity, clinical signs, investigations, comorbidities, and psychological factors (Supplementary Table 3). Eighteen prognostic factors were assessed in at least two studies (Table 6). The factors that were reported to have an association with deteriorating pain or function in any study were as follows: disease duration of greater than 5 years [34]; high hand load for leisure [36]; low baseline grip strength [37]; high baseline level of pain and functional difficulty [38,39,46]; a greater number of painful hand joints [39] or erosive hand joints [38], a single comorbidity of heart disease [30] or diabetes [42] multiple co-morbidities [42], certain illness perceptions [28] and the use of certain coping strategies [45]. Additionally, some studies identified prognostic factors for different outcomes, including ethnicity with an increase in the number of symptomatic joints [48], and the presence of coexisting knee OA [34] to worsening global assessment of OA. Lack of deterioration was associated in single studies with older age at the onset of osteoarthritis [46] and the use of non-steroidal anti-inflammatory medications [34].

**Table 6.** Prognostic factors for deterioration or poor outcome of hand pain or function assessed in at least two studies.

Prognostic factor	Study: author year	Follow up (years)	Risk of bias	Definition of progression	Results of univariable/multivariable analyses	Univariable/ multivariable analyses	Association with OA prognosis
**Demographic** and **Social**							
*Sex*	Dieppe [34]	8	HIGH	Patient reported outcomes for pain and overall condition (better, same, worse)	Descriptive data (chi squared and significance at 5% level) NS	Univariable analysis	(o)
	Vanhaverbeke [46]	9.7	HIGH	AUSCAN function (0–90), change ≥ 7.2 or FIHOA (0–30), change ≥ 1.8.	OR (95% CI) 1.94 (0.32,11.9) *p* = 0.0472	Univariable analysis	(o)
	Neuprez [38]	2	HIGH	AUSCAN (0–300) change > MCID 15, AUSCAN pain (0–100) >8 or AUSCAN function (0–100) >4	Not statistically significant estimate not provided.	Multivariable analysis	(o)
	Snyder [48]	12	HIGH	Change in number of symptomatic joints (0–30), (pain/ tenderness and radiographic OA in the same joint)	OR (95% CI) 1.25 (0.72,2.16)	Multivariable: age, sex, race, education, BMI, weight gain >5%, HOA baseline values (KLG, nos radiographic and symptomatic joints)	(o)
*Occupation*	Dieppe [34]	8	HIGH	patient reported outcomes and overall condition (better, same, worse)	Descriptive data (chi squared and significance at 5% level) NS	Univariable analysis	(o)
	Ding [36]	5	HIGH	Incident/persistent pain: ≥1 joint thumb, index, or middle finger or separately ring or little finger, vs reference group of no pain at either time point radial or separately ulnar digits	PR (95% CI) (Dentist vs teacher [teacher = ref]) 1.34(0.89, 2.02)	Multivariable: age, occupation, BMI, smoking, leisure hand activity, work status, radiographic joint OA	(o)
*Age*	Dieppe [34]	8	HIGH	patient reported outcomes for pain and overall condition (better, same, worse)	Descriptive data (chi squared and significance at 5% level) NS	Univariable analysis	(o)
	Vanhaverbeke [46]	9.7	HIGH	AUSCAN function (0–90), change ≥ 7.2 or FIHOA (0–30), change ≥ 1.8.	OR (95% CI) 0.93 (0.87, 0.99) *p* = 0.022 (greater age at onset was protective)	Univariable analysis	(–)
	Ding [36]	5	HIGH	Incident/persistent pain: ≥1 joint thumb, index or middle finger or separately ring or little finger, vs reference group of no pain at either time point radial or separately ulnar digits	PR (95% CI) 1.01(0.96, 1.08)	Multivariable: age, occupation, BMI, smoking, leisure hand activity, work status, radiographic joint	(o)
	Neuprez [38]	2	HIGH	AUSCAN (0–300) change > MCID 15, AUSCAN pain (0–100) >8 or AUSCAN function (0–100) >4	Not statistically significant, estimate not provided.	Multivariable analysis	(o)
*Smoker*	Ding [36]	5	HIGH	Incident/persistent pain: ≥1 joint thumb, index or middle finger or separately ring or little finger, vs reference group of no pain at either time point radial or separately ulnar digits	PR (95% CI) smoking ever 0.97(0.62, 1.54)	Multivariable: age, occupation, BMI, smoking, leisure hand activity, work status, radiographic joint OA	(o)
	Courties [30]	2.6	HIGH	Deterioration from baseline scores AUSCAN/FIHOA	OR (95% CI) 1.41 (0.39, 5.09) *p* = 0.59	Univariable analysis	(o)
	Neuprez [38]	2	HIGH	AUSCAN (0–300) change > MCID 15, AUSCAN pain (0–100) >8 or AUSCAN function (0–100) >4	Not statistically significant, estimate not provided.	Multivariable analysis	(o)
*Alcohol consumption*	Courties [30]	2.6	HIGH	Deterioration from baseline scores AUSCAN/FIHOA	OR (95% CI) 1.01 (0.64, 1.9)	Univariable analysis	(o)
	Neuprez [38]	2	HIGH	AUSCAN (0–300) change > MCID 15, AUSCAN pain (0–100) >8 or AUSCAN function (0–100) >4	Not statistically significant, estimate not provided.	Multivariable analysis	(o)
*Menopausal status*	Courties [30]	2.6	HIGH	Deterioration from baseline scores AUSCAN/FIHOA	OR (95% CI) 0.75 (0.4,1.44) *p* = 0.39	Univariable analysis	(o)
	Neuprez [38]	2	HIGH	AUSCAN (0–300) change > MCID 15, AUSCAN pain (0–100) >8 or AUSCAN function (0–100) >4	Not statistically significant, estimate not provided.	Multivariable analysis	(o)
*BMI*	Courties [30]	2.6	HIGH	Deterioration from baseline scores AUSCAN/FIHOA	OR (95% CI) 1.38 (0.79, 2.39) *p* = 0.25	Univariable analysis	(o)
	Dieppe [34]	8	HIGH	patient reported outcomes for pain and overall condition (better, same, worse)	Descriptive data (chi squared and significance at 5% level) NS	Univariable analysis	(o)
	Vanhaverbeke [46]	9.7	HIGH	AUSCAN function (0–90), change ≥ 7.2 or FIHOA (0–30), change ≥ 1.8.	OR (95% CI) 0.99(0.93, 1.05) *p* = 0.8	Univariable analysis	(o)
	Ding [36]	5	HIGH	Incident/persistent pain: ≥1 joint thumb, index, or middle finger or separately ring or little finger, vs reference group of no pain at either time point radial or separately ulnar digits	PR (95% CI) 1.23(0.86, 1.76)	Multivariable: age, occupation, BMI, smoking, leisure hand activity, work status, radiographic joint OA	(o)
	Siviero [37]	1.5	LOW	MCID score of >4 AUSCAN function	RR (95% CI) 0.95 (0.79, 1.15) *p* = 0.62	Multivariable: sex, age, country, education level	(o)
	Neuprez [38]	2	HIGH	AUSCAN (0–300) change > MCID 15, AUSCAN pain (0–100) >8 or AUSCAN function (0–100) >4	Not statistically significant, estimate not provided.	Multivariable analysis	(o)
*Use of analgesics* */NSAIDs*	Siviero [37]	1.5	LOW	MCID score of >4 AUSCAN function	RR (95% CI) 1.01 (0.83, 1.21) *p* = 0.96	Multivariable: sex, age, country, education level	(o)
	Dieppe [34]	8	HIGH	patient reported outcomes and overall condition (better, same, worse)	Descriptive data (chi squared and significance at 5% level). Patients using NSAIDs were more likely to report improvement (24%) than those not using NSAIDs (12%) *p* = 0.017	Univariable analysis	(–)
**Symptom severity**							
*Baseline self-reported pain* and *function*	Bijsterbosch [39]	6	LOW	Poor outcome not fulfilling PASS for AUSCAN (>8.2, 16.1)	RR (95% CI) ∞ Pain: pain > 8: 5.74(4.38, 6.65) function >16: 2.57(1.26,4.13); Function: pain >8: 3.56(1.63,5.83), function >16:6.88(5.3, 7.9).	Multivariable: baseline scores of the clinical outcome measure, follow-up time, family effects	(+)
	Siviero [37]	1.5	LOW	MCID score of >4 AUSCAN function	RR (95% CI) pain score ≥5 1.11 (0.89, 1.37) *p* = 0.35	Multivariable: sex, age, country, education level	(o)
	Vanhaverbeke [46]	9.7	HIGH	AUSCAN function (0–90), change ≥ 7.2 or FIHOA (0–30), change ≥ 1.8.	OR (95% CI) VAS >33mm 1(0.34, 2.94) *p* = 0.995 FIHOA 0.85(0.77, 0.94) *p* = 0.002	Univariable analysis	(o) pain
	Courties [30]	2.6	HIGH	Deterioration from baseline scores AUSCAN/FIHOA	OR (95% CI) 0.956 (0.88, 1.038) *p* = 0.28	Multivariable: no adjustment, FIHOA, CHD and depression	(o)
	Neuprez [38]	2	HIGH	AUSCAN (0–300) change > MCID 15, or AUSCAN function (0–100) >4	OR (95% CI) AUCAN score <74.5 1.02 (1.01,1.03) *p* < 0.01. AUSCAN pain score <47 OR 1.03 (1.01,1.04) *p* < 0.01, AUSCAN function score <56 OR 1.02 (1.01,1.03) *p* < 0.01	Multivariable analysis	(+)
**Investigations**							
*Imaging (radiograph/ MRI severity)*	Bijsterbosch [39]	6	LOW	Poor outcome not fulfilling PASS for AUSCAN (>8.2, 16.1)	RR (95% CI) ∞ pain osteophytes >11: 1.22 (0.77,1.73) JSN >22: 1.02(0.64,1.46) TBOA 0.92(0.56, 1.38) Function: osteophytes >11:0.72 (0.4,1.19) JSN >22:0.79 (0.45, 1.23)	Multivariable: baseline scores of the clinical outcome measure, follow-up time, family effects	(o)
	Courties [30]	2.6	HIGH	Deterioration from baseline scores FIHOA	radiographic scores OR (95% CI) 0.99 (0.97, 1.01) p 0.24	Univariable analysis	(o)
	Neuprez [38]	2	HIGH	AUSCAN (0**–**300) change > MCID 15, AUSCAN pain (0–100) >8 or AUSCAN function (0–100) >4	Not statistically significant, estimate not provided.	Multivariable analysis	(o)
	Van Beest [49]	2	HIGH	increased thumb base pain on palpation (0–3)	(osteophytes) ORs (95% CI) 1.73 (0.73,4.1)	Multivariable analysis adjustments for other imaging scores.	(o)
*Erosive OA (number of baseline E joints* and *R joints)*	Vanhaverbeke [46]	9.7	HIGH	AUSCAN function (0–90), change ≥ 7.2 or FIHOA (0–30), change ≥ 1.8.	OR (95% CI) Function total R joints 1.06 (0.85,1.32) *p* = 0.617 total E joints 1.15 (0.86,1.54) *p* = 0.339	Univariable analysis	(o)
	Neuprez [38]	2	HIGH	AUSCAN (0–300) change > MCID 15, AUSCAN pain (0-100) >8 or AUSCAN function (0–100) >4	OR (95% CI) ∞ Worsening function total ≥4 R and E joints 2.26 (1.07,4.78) *p* = 0.03	Multivariable analysis	(+)
**Clinical Signs**							
*Number of painful joints*	Bijsterbosch [39]	6	LOW	Poor outcome not fulfilling PASS for AUSCAN (>8.2, 16.1)	RR (95% CI) Poor outcome pain >8: 2.11 (1.25, 3.08) poor outcome function >8: 2.39 (1.47,3.37)	Multivariable: baseline scores of the clinical outcome measure, follow-up time, family effects	(+)
	Neuprez [38]	2	HIGH	AUSCAN (0–300) change > MCID 15, AUSCAN pain (0–100) >8 or AUSCAN function (0–100) >4	Not statistically significant, estimate not provided.	Multivariable analysis	(o)
*Number of painful nodes* *(Heberdens* and *Bouchards)*	Bijsterbosch [39]	6	LOW	Poor outcome not fulfilling PASS for AUSCAN (>8.2, 16.1)	RR (95% CI) ∞ Poor outcome for pain >11: 1.44 (0.91,2.02) poor outcome function >11: 0.98 (0.56,1.57)	Multivariable: baseline scores of the clinical outcome measure, follow-up time, family effects	(o)
	Neuprez [34]	2	HIGH	AUSCAN (0–300) change > MCID 15, AUSCAN pain (0–100) >8 or AUSCAN function (0–100) >4	Not statistically significant, estimate not provided.	Multivariable analysis	(o)
*Grip strength*	Vanhaverbeke 2020 [46]	9.7	HIGH	AUSCAN function (0–90), change ≥ 7.2 or FIHOA (0–30), change ≥ 1.8.	OR (95% CI) 0.9 (0.9,1.06) *p* = 0.586	Univariable analysis	(o)
	Siviero [37]	1.5	LOW	MCID score of >4 AUSCAN function	RR (95% CI) Grip [ref >35kg] < 20.5 kg 1.74: (1.17, 2.59) *p* = 0.006 20.5–26.75 kg: 1.78 (1.23,2.58 *p* = 0.002 26.75–35kg: 1.61(1.17,2.24) *p* = 0.04	Multivariable: sex, age, country, education level	(+)
**Co-morbidities**							
*Hypertension*	Dieppe [34]	8	HIGH	patient reported outcomes and overall condition (better, same, worse)	Descriptive data (chi squared and significance at 5% level) NS	Univariable analysis	(o)
	Courties [30]	2.6	HIGH	Deterioration from baseline scores AUSCAN/FIHOA	OR (95% CI) 1.1 (0.64,1.9) *p* = 0.72	Univariable analysis	(o)
	Neuprez [38]	2	HIGH	AUSCAN (0–300) change > MCID 15, AUSCAN pain (0–100) >8 or AUSCAN function (0–100) >4	Not statistically significant, estimate not provided.	Multivariable analysis	(o)
*Coronary Heart Disease*	Courties [30]	2.6	HIGH	Deterioration from baseline scores AUSCAN/FIHOA	OR (95% CI) 2.91 (1.02,8.26) *p* = 0.045	Multivariable: not specified which adjustments were made.	(+)*
	Siviero [37]	1.5	LOW	MCID score of >4 AUSCAN function.	RR (95% CI) 1.05 (0.88,1.25) *p* = 0.62	Multivariable: sex, age, country, education level	(o)
	Scherzer [42]	12	HIGH	Transitions from AUSCAN pain categories ≤6 ‘low’ or AUSCAN >6 ‘high’ AUSCAN function sub score ≤9 was defined as ‘better function’ AUSCAN function sub score >9 was defined as ‘worse function.’	HR (95% CI) 0.99 (0.63,1.55)	Multivariable: age, sex, race, education level, BMI, symptomatic KOA, NSAID use	(o)
	Neuprez [38]	2	HIGH	AUSCAN (0–300) change > MCID 15, AUSCAN pain (0–100) >8 or AUSCAN function (0–100) >4	Not statistically significant, estimate not provided.	Multivariable analysis	(o)
*Diabetes*	Courties [30]	2.6	HIGH	Deterioration from baseline scores AUSCAN/FIHOA	OR (95% CI) 0.84 (0.35,2.01) *p* = 0.69	Univariable analysis	(o)
	Scherzer [42]	12	HIGH	Transitions in AUSCAN pain categories ≤6 ‘low’ or AUSCAN >6 ‘high’ AUSCAN function sub score ≤9 was defined as ‘better function’ AUSCAN function sub score >9 was defined as ‘worse function.’	No. with diabetes/number without, HR (95% CI) worsening AUSCAN pain: 11/25 5.08 (1.38,18.77)	Multivariable: age, sex, race, education level, BMI, symptomatic KOA, NSAID use	(+)
	Siviero [37]	1.5	LOW	MCID score of >4 AUSCAN function.	Adj RR (95% CI) 0.86 (0.65,1.12) *p* = 0.25	Multivariable: sex, age, country, education level	(o)
	Neuprez [38]	2	HIGH	AUSCAN (0–300) change > MCID 15, AUSCAN pain (0–100) >8 or AUSCAN function (0–100) >4	Not statistically significant, estimate not provided.	Multivariable analysis	(o)
**Psychological**							
*Depression*	Siviero [37]	1.5	LOW	MCID score of >4 AUSCAN function	RR (95% CI) *p* value 1.14 (0.90, 1.44) *p* = 0.29	Multivariable: sex, age, country, education level	(o)
	Courties [30]	2.6	HIGH	Deterioration from baseline scores AUSCAN/FIHOA	OR (95% CI) 0.44 (0.18,1.05) *p* = 0.06	Univariate analysis	(o)
	Neuprez [38]	2	HIGH	AUSCAN (0–300) change > MCID 15, AUSCAN pain (0–100) >8 or AUSCAN function (0–100) >4	Not statistically significant, estimate not provided.	Multivariable analysis	(o)

Key: NS Statistically non-significant, MCII minimum clinically important improvement, PASS patient acceptable symptom state, OR odds ratio, RR risk ratio, HR hazard ratio, PR prevalence ratio, CI confidence interval, Adj adjusted analysis, unadj unadjusted analysis, NA not applicable, NS not significant, CORS coping with rheumatic stressors survey, NSAIDs non-steroidal anti-inflammatory drugs, FIHOA functional index for hand OA, AUSCAN Australian Canadian hand osteoarthritis index, BMI body mass index, KOA knee osteoarthritis, KLG Kellgren**–**Lawrence radiographic grading system, nos numbers of, MCID minimal clinically important difference, E Erosive, R Remodelled.

*Multivariate analyses where a significant direction effect is reported.

∞Indicates estimates for the highest tertile of each prognostic factor values are shown.

The GRADE assessment found that the prognosis of persistent or worsening pain and worsening function conclusions were limited by having only single studies for a given factor (providing only exploratory evidence for their value as a prognostic factor). Where prognostic factors were assessed in multiple studies, there was variation in the direction of the effects, follow-up periods, outcome assessments, data analysis, and data presentation. These factors affected the consistency and precision of the judgements. Finally, most judgements were downgraded for quality because most studies had a high risk of bias. Moderate-level evidence of persistent or worsening hand pain was found for the presence of diabetes as a prognostic factor. Low-level evidence was found for the following prognostic factors: self-reported baseline pain, self-reported baseline function, age, and number of painful hand joints. For the outcome of worsening hand function moderate level evidence was found for the prognostic factor of self-reported pain, and low-level evidence for the prognostic factors of: self-reported function; number of painful hand joints; number of erosive hand joints; grip strength; presence of coronary heart disease; income; and certain illness perceptions (Table 6; Supplementary Table 4).

## Discussion

This review summarizes and critically appraises evidence regarding the course and prognosis of symptomatic hand OA including subtypes of TBOA, nodal OA, and erosive OA). Evidence from this review suggests that individuals with hand OA experience different symptom courses, ranging from improvement to deterioration. On average, there is little change in the hand pain experienced over both short and long time periods, and hand function and grip strength do deteriorate, but, on average, by small amounts. Forty-six prognostic factors of persistent or worsening hand pain and worsening hand function have been examined, but the majority have only been investigated in a single study or where replicated studies show contradictory results. However, our evidence indicates that deterioration in hand pain and function is associated with diabetes and baseline pain severity, respectively.

The different symptom courses of hand and thumb pain are evidenced by the varying proportions of individuals whose pain worsened and those whose pain improved in nine different studies, but also by the wide standard deviations around the mean for baseline, follow-up, and change scores. There is no certainty regarding the estimates for the proportions with worsening pain over the short and long term due to variations in the outcome measures used and different definitions of worsening hand pain. Additionally, the outcome of pain in hand OA can be assessed in different ways, which do not necessarily correlate, but are each valid. For example, the number of tender joints on palpation may be affected by inflammation, but it cannot be used interchangeably with patient reported pain severity. The latter may relate more to central pain sensitization, as well as coping skills and illness perception [54].

In comparison, moderate evidence was found for changes in hand function over the longer term, which, on average, deteriorated by a small amount. Again, the evidence from the studies suggests that this was variable among individuals, with approximately 50% who experienced deterioration. There was also moderate evidence that grip strength deteriorated over time, and the greater the length of time, the greater the deterioration that was observed. Aging could explain some of this change [55], but in this review, it was not possible to consider strength relative to expected age-related norms. However, grip strength in hand OA is reduced early in the radiographic disease course [56] and may be most affected in TBOA [56–58]. In this review, the four-to-seven-year average changes in grip strength did not meet the 0.84–1.12 kg minimum clinically important difference [59] but over 10 years the deterioration exceeded this and would impact on an individual’s hand function [60], suggesting that strengthening grip could be an important longer-term treatment target, especially in early onset hand and TBOA.

Few studies have examined TBOA separately from hand OA. Two low-quality studies were found in one cohort assessing TBOA [33,40], but the data at baseline represented only those with early radiographic disease and excluded those who underwent surgery during follow-up. It is uncertain whether the improved symptomatic course observed in these studies may be representative of TBOA in general. Another study assessed changes in pain on thumb base palpation, finding over two years that 16% improved and 11% worsened [49], if pain on palpation corresponded to the pain levels experienced, then the proportions might suggest that fewer participants experience worsening of their TBOA pain than with hand OA (25%) [38]. However, two studies presented data for hand OA subsets separately; one community population found no significant differences between the subsets for hand pain and function at 3-years adjusted for baseline [7], while the other found that TBOA was associated with a reduction in grip strength over time [16], suggesting that TBOA impacts not only pinch and dextrous tasks but also whole hand grip strength more than other subgroups. While this review is interested in progression, it is important to point out that even with little progression, studies suggest that pain levels can be high for some individuals and remain high over time. One study found that 54.4% of patients with clinical and radiographic TBOA had symptoms above the patient acceptable symptom state [39].

To date, only one systematic review has examined the prognostic factors for progression of hand OA [6]. This study adds to the previous systematic review by summarizing the data from more recent studies and is focused specifically on the course of symptoms and associated prognostic factors. As with the previous literature, most of the studies can be viewed as exploratory studies that identify new and promising factors, which require further confirmatory research. Four studies were judged by the reviewers as confirmatory studies [28,29,42,45], which examined the added prognostic value of an identified factor after adjusting for established, prognostic factors [61]. From a broad range of factors assessed, this review identified low evidence for a relationship between deteriorating hand pain or hand function and five prognostic factors (baseline function, negative illness perceptions, the number of painful hand joints, number of erosive hand joints, and the presence coronary heart disease). Two prognostic factors were assessed as having a moderate level of evidence: diabetes for persistent or worsening hand pain, and high levels of baseline hand pain as prognostic factors for poor functional outcome. However, all studies assessing both factors differed in the direction of the effect of this relationship. The different analytical techniques and assumptions used are a possible cause for this inconsistency, for example, the use of an unadjusted model in one study and a very different cut-off point for high baseline level of hand pain in all studies.

A better understanding of the characteristics that indicate which patients will deteriorate or will not respond to current treatment could be used to improve pathways of care and develop new treatments targeting modifiable prognostic factors. Passive coping strategies and negative illness perceptions are potentially modifiable and are not current treatment targets for hand and TBOA. Self-reported pain severity is known to be strongly related to psychological factors [62], suggesting that new treatments could be considered to improve pain coping skills and illness perceptions.

### Limitations

This systematic review had several limitations. There was large heterogeneity in the data studied; hence, this review provides the best evidence narrative summary, rather than a pooled summary estimate meta-analysis. Future studies would benefit from describing their populations by hand OA subtype, and assessing outcomes according to subtype, as the differing subtypes may have differing symptomatic course and prognostic factors. Additionally, future research would benefit from an agreed definition of worsening or improving symptoms, a consistent approach to presenting outcome data, and a standardized length of follow-up for short- and long-term studies. Additionally, the quality of prognostic factor studies could be improved by collecting and providing data on study participation (source population and adequate participation by eligible individuals) and confounding factors (details on the factors assessed, their measurement method, and how they have been accounted for in the analysis). This review intentionally included a broad spectrum of hand OA in terms of the diagnostic criteria used and the setting of the research. This means that all levels of severity of condition are included, but this could be considered a limitation, as the stage of advancement at baseline or severity of symptoms may impact the symptomatic course.

### Future research and clinical implications

This review suggests that the course of symptoms in hand and TBOA is variable between individuals. Further research is required into the prognostic factors that might indicate those at risk of persistent pain and poor function as this review found either limited evidence or contradictory results.

This review demonstrates that grip strength and hand function in people with hand and thumb base OA does deteriorate by small but increasing amounts over time. It would be important to consider this as a potential target for interventions and when looking at longer-term results of interventions, e.g. over three years. Halting deterioration by maintaining strength and hand function should be seen as a positive outcome, alongside any gains achieved.

Results from two randomized controlled trials [63,64], suggest the outcome of non-operative care is also variable. Prognostic factors for the outcomes of symptom course and response to treatment may overlap. The prognostic factors identified from this review for symptom course should also be explored as potential predictors of treatment effect, which will require analysis of evidence from randomised trials of intervention for hand OA.

## Conclusion

This review summarizes and critically appraises evidence regarding the course and prognosis of symptomatic hand OA and thumb base OA. Evidence from this review suggests that, on average, there is little change in the hand pain experienced with hand OA over both short and long periods, whereas hand function and grip strength do deteriorate but, on average, by small amounts. There is evidence to suggest that there are differing symptom courses in hand OA, but there is limited or contradictory evidence of prognostic factors that might indicate a worsening trajectory. Prognostic factors that may indicate a higher likelihood of increasing hand pain and worsening hand function are related to general health, psychological factors, and perceived severity of symptoms. Further research is needed to confirm the predictive performance of these factors and broaden our understanding of the prognostic factors for worsening pain in hand and thumb base OA.

## Supplementary Material

Supplemental Material

## Data Availability

The authors confirm that the data supporting the findings of this study are available in the article and its supplementary materials.
