# Supplementary material for: The prognosis of pain and function in people with hand and thumb base osteoarthritis: a systematic review
Source: Ann Med. 2025 Jul 25;57(1):2532113. doi: 10.1080/07853890.2025.2532113 (PMC12302428; doi:10.1080/07853890.2025.2532113)
Supplement: Supplemental Material [file IANN_A_2532113_SM8096.pdf]

**Supplementary Table 1.1 The full search strategy for Ovid Medline Search terms**
(exp Osteoarthritis/ or osteoarthr*.ti,ab.)<br>AND<br>((hand* or finger* or wrist* or thumb* or metacarp* or trapeziometacarpal joint or carpometacarpal joint or hand deformities or hand joints or hand bones).mp.<br>OR (exp Metacarpus/ or exp Hand Bones/ or exp Hand/ or exp Fingers/ exp Hand/ or exp Hand Deformities/ or exp Hand Bones/ or exp Hand Joints/ or exp Hand Deformities, Acquired/ or exp Wrist Joint/ or exp Carpal Bones/ or exp Carpal Joints/ exp Carpometacarpal Joints/ or exp Thumb/ or exp Metacarpophalangeal Joint/))<br>AND<br>((disease* or natural or clinical) adj2 (history or course or progress* or trajector*)).mp. or (outcome.mp.) or (exp Treatment Outcome/) OR (cohort stud* or prospective stud* or longitudinal stud* or epidemiological stud*).mp or (Risk Factor.mp.) or (exp Risk Factors/) or (disease progression.mp.) or (exp Disease Progression/) or prognosis.mp. or (exp Prognosis/) or (prognostic factor*.mp.) or predictor.mp.)

**Supplementary Table 1.2 The full search strategy for EBSCO CINAHL Search terms**
((TI (osteoarth*) OR AB (osteoarth*) OR (MH "Osteoarthritis"))<br>AND<br>((MH "HAND+" or "FINGERS+" OR "HAND DEFORMITIES+" OR "HAND JOINTS+") OR (MH wrist joint OR carpal joints or carpometacarpal joints or finger joint or metacarpophalangeal joint))<br>AND<br>(TX (disease* OR natural or clinical) N2 (history or course or progress*) OR (AB TI (disease* OR natural or clinical) N2 (history or course or progress*) or (TX outcome or "risk factor" or epidemiological research or cohort or prospective* or longitudinal)))

**Supplementary Table 1.3 The full search strategy for Ovid EMBASE Search terms**
((osteoarthr*.mp.) OR (exp Osteoarthritis/))<br>AND<br>((hand* or finger* or wrist* or thumb* or metacarp* or trapeziometacarpal joint or carpometacarpal joint or hand deformities or hand joints or hand bones).mp. OR (exp metacarpal bone/ or exp hand bone/ or exp hand osteoarthritis/ or exp hand disease/ or exp hand/ or exp hand joint/ or exp finger/ or exp finger joint/ or exp Wrist/ or exp Carpal Bone/ or exp Carpal Joint/ or exp Carpometacarpal Joint/ or exp Thumb/ or exp Metacarpophalangeal Joint/))<br>AND<br>(((disease* or natural or clinical) adj2 (history or course or progress* or trajector*)).mp. OR outcome.mp. or (exp Treatment Outcome/) OR (Risk Factor.mp.) or (exp risk factor/) or (cohort stud* or prospective stud* or longitudinal stud* or epidemiological stud*).mp OR (disease progression).mp. OR (prognosis.mp.) or (exp prognosis/) OR (prognostic factor*).mp. OR (predictor.mp.))

**Supplementary Table 1.4 The full search strategy for AGELINE EBSCO Search terms**
AB osteoarthr* OR TI osteoarthr* OR SU osteoarthr*<br>AND<br>AB ((hand* or finger* or wrist* or thumb* or metacarp* or trapeziometacarpal joint or carpometacarpal joint or hand deformities or hand joints or hand bones)) OR TI ((hand* or finger* or wrist* or thumb* or metacarp* or trapeziometacarpal joint or carpometacarpal joint or hand deformities or hand joints or hand bones)) OR SU ((hand* or finger* or wrist* or thumb* or metacarp* or trapeziometacarpal joint or carpometacarpal joint or hand deformities or hand joints or hand bones))

**Supplementary Table 1.5 The full search strategy for PROQUEST PSYCINFO Search terms**
ti(osteoarth* OR arthriti* OR arthrosis OR athrotic) OR ab(osteoarth* OR arthriti* OR arthrosis OR athrotic) OR su(OSTEOARTHRITIS OR ARTHRITIS)<br><br>AND<br><br>ti(hand* OR finger* OR wrist* OR metacarp* OR trapeziometacarpal joint OR carpometacarpal joint OR hand deformities OR hand joints OR hand bones) OR ab(hand* OR finger* OR wrist* OR metacarp* OR trapeziometacarpal joint OR carpometacarpal joint OR hand deformities OR hand joints OR hand bones) OR su(HAND BONES OR METACARPUS OR FINGERS OR HAND OR HAND BONES OR HAND DEFORMITIES OR HAND JOINTS OR WRIST JOINT OR CARPAL JOINTS OR CARPOMETACARPAL JOINTS OR FINGER JOINT OR METACARPOPHALANGEAL JOINT)<br><br>AND<br><br>(disease OR natural OR clinical) adj2 (history OR course OR progress) OR su(DISEASE PROGRESSION OR PROGNOSIS) OR (outcome OR "risk factor" OR epidemiological research OR cohort OR prospective OR longitudinal)

Commented [VJ1]: Suzanne This search wasn't written in the same way as the others, can you check I have put it in correctly thanks

ALSO I don't have a separate strategy for AMED does this mean it follows one of the others exactly - can you tell me which?

Perhaps just cast your eyes over all of them re spacing caps etc. Thanks

Supplementary Table 1.6 The full search strategy for Ovid AMED Search terms
((osteoarthr*.mp.) OR (exp Osteoarthritis/))<br><br>AND<br><br>((hand* or finger* or wrist* or thumb* or metacarp* or trapeziometacarpal joint or carpometacarpal joint or hand deformities or hand joints or hand bones).mp. OR (exp metacarpal bone/ or exp hand bone/ or exp hand osteoarthritis/ or exp hand disease/ or exp hand/ or exp hand joint/ or exp finger/ or exp finger joint/ or exp Wrist/ or exp Carpal Bone/ or exp Carpal Joint/ or exp Carpometacarpal Joint/ or exp Thumb/ or exp Metacarpophalangeal Joint/))<br><br>AND<br><br>(((disease* or natural or clinical) adj2 (history or course or progress* or trajector*)).mp. OR outcome.mp. or (exp Treatment Outcome/) OR (Risk Factor.mp.) or (exp risk factor/) or (cohort stud* or prospective stud* or longitudinal stud* or epidemiological stud*).mp OR (disease progression).mp. OR (prognosis.mp.) or (exp prognosis/) OR (prognostic factor*).mp. OR (predictor.mp.))

Supplementary Table 2. Criteria for GRADE Assessment for Prognosis

For prognosis related to specific outcomes, evidence from at least two well-conducted cohort studies of sufficient sample sizes and with consistent findings was considered high-quality evidence on overall prognosis or prognostic factors. The evidence was then assessed for down grading on the following basis:			
Risk of bias	*Inconsistency*	*Imprecision*	*Indirectness*
Downgrade if less than 2 cohorts at low risk of bias, or >50% of the cohorts were considered high risk of bias.	Downgrade if studies demonstrate clinical or methodological heterogeneity (e.g., use of very different outcome measures or length of follow-up). Or point estimates and confidence intervals of the outcome don't overlap or prognostic factor association with the outcome vary in direction (for example, some effects appear protective whereas others show risk).	Downgrade with a single cohort and/or majority of studies (had sample size smaller than 200).	Downgrade if the results considered for the outcome across included studies relates only to a subset of the population of interest (e.g., proportions who improved/ deteriorated)
For prognostic factors all factors were assessed if they demonstrated a positive relationship with outcome, in a single study. Indirectness according to the definition above did not apply to prognostic factors, so downgrading was assessed for risk of bias, inconsistency, and imprecision only.			

731 **Supplementary Table 3. All prognostic factors assessed in the reviewed literature.**

Prognostic factors	Study	Follow up (years)	Risk of bias	Definition of progression	Results of univariable/multivariable analyses	Univariable/ multivariable analyses	Association with OA prognosis
**Demographic & Social**							
Sex	Dieppe 2000[34]	8	HIGH	patient reported outcomes (better, same, worse)	Descriptive data (chi squared and significance at 5% level) NS	Univariable analysis	(o)
	Vanhaverbeke 2020[46]	9.7	HIGH	AUSCAN function (0–90), change ≥ 7.2 or FIHOA (0–30), change ≥ 1.8.	OR (95% CI) 1.94 (0.32,11.9) p=0.0472	Univariable analysis	(o)
	Neuprez 2021[38]	2	HIGH	AUSCAN (0-300) change >MCID 15, AUSCAN pain (0-100) >8 or AUSCAN function (0-100) >4	Not statistically significant, estimate not provided.	Multivariable analysis	(o)
	Snyder 2020[48]	12	HIGH	Change in number of symptomatic joints (0-30), (pain/ tenderness and radiographic OA in the same joint)	OR (95% CI) 1.25 (0.72,2.16)	Multivariable: age, sex, race, education, BMI, weight gain > 5%, HOA baseline values (KLG, nos radiographic and symptomatic joints)	(o)
Ethnicity/race	Snyder 2020[48]	12	HIGH	Change in number of symptomatic joints (0-30), (pain/ tenderness and radiographic OA in the same joint)	White vs African American (ref) OR 1.96 (1.01, 3.83)	Multivariable: age, sex, race, education, BMI, weight gain > 5%, HOA baseline values (KLG, nos radiographic and symptomatic joints)	(+)
Occupation	Dieppe 2000[34]	8	HIGH	patient reported outcomes (better, same, worse)	Descriptive data (chi squared and significance at 5% level) NS	Univariable analysis	(o)
	Ding 2011[36]	5	HIGH	Persistent pain: ≥1 joint thumb, index or middle finger or separately ring or little finger, vs reference group of no pain at either time point radial or separately ulnar digits	PR (95% CI) (Dentist vs teacher [teacher = ref]) 1.34 (0.89,2.02)	Multivariable: age, occupation, BMI, smoking, leisure hand activity, work status, radiographic joint OA	(o)

Income	Siviero 2020[37]	1.5	LOW	MCID score of >4 AUSCAN function	RR (95% CI) 0.6 (0.36,0.99) p=0.05	Multivariable: sex, age, country, education level	(-)
Social isolation	Siviero 2020[37]	1.5	LOW	MCID score of >4 AUSCAN function	RR (95% CI) 1.03 (0.84,1.26) p=0.81	Multivariable: sex, age, country, education level	(o)
Physical activity	Siviero 2020[37]	1.5	LOW	MCID score of >4 AUSCAN function	RR (95% CI) < 105 min/day 0.92 (0.73-1.16) p=0.81	Multivariable: sex, age, country, education level	(o)
Medical/social care	Siviero 2020[37]	1.5		MCID score of >4 AUSCAN function	RR (95% CI) 1.16 (0.92,1.46) p=0.21	Multivariable: sex, age, country, education level	(o)
High hand leisure time	Ding 2011[36]	5	HIGH	Incident/persistent pain: ≥1 joint thumb, index or middle finger or separately ring or little finger, vs reference group of no pain at either time point radial or separately ulnar digits.	PR (95% CI) right: 1.37 (0.9,2.08) left: 1.68(1.00,2.83)	Multivariable: age, occupation, BMI, smoking, leisure hand activity, work status, radiographic joint OA	(+) left
Family history of OA	Dieppe 2000[34]	8	HIGH	patient reported outcomes (better, same, worse)	Descriptive data (chi squared and significance at 5% level) NS	Univariable analysis	(o)
Family History of Hand OA	Neuprez 2021[38]	2	HIGH	AUSCAN (0-300) change >MCID 15, AUSCAN pain (0-100) >8 or AUSCAN function (0-100) > 4	Not statistically significant, estimate not provided.	Multivariable analysis	(o)
Disease duration	Dieppe 2000[34]	8	HIGH	patient reported outcomes (better, same, worse)	Average of 5 years longer disease duration in those reporting a worsening outcome (p=0.007)	Univariable analysis	(+)
Age	Dieppe 2000[34]	8	HIGH	patient reported outcomes (better, same, worse)	Descriptive data (chi squared and significance at 5% level) NS	Univariable analysis	(o)
	Vanhaverbeke 2020[46]	9.7	HIGH	AUSCAN function (0–90), change ≥ 7.2 or FIHOA (0–30), change ≥ 1.8.	OR (95% CI) 0.93 (0.87,0.99) p=0.022 (greater age at onset was protective)	Univariable analysis	(-)
	Ding 2011[36]	5	HIGH	Incident/persistent pain: ≥1 joint thumb, index or middle finger or separately ring or little finger, vs reference group of no pain at either time point radial or separately ulnar digits.	PR (95% CI) 1.01 (0.96,1.08)	Multivariable: age, occupation, BMI, smoking, leisure hand activity, work status, radiographic joint	(o)

	Neuprez 2021[38]	2	HIGH	AUSCAN (0-300) change >MCID 15, AUSCAN pain (0-100) >8 or AUSCAN function (0-100) > 4	Not statistically significant, estimate not provided.	Multivariable analysis	(o)
Smoker	Ding 2011[36]	5	HIGH	Incident/persistent pain: ≥1 joint thumb, index or middle finger or separately ring or little finger, vs reference group of no pain at either time point radial or separately ulnar digits.	PR (95% CI) smoking ever 0.97 (0.62,1.54)	Multivariable: age, occupation, BMI, smoking, leisure hand activity, work status, radiographic joint OA	(o)
	Neuprez 2021[38]	2	HIGH	AUSCAN (0-300) change >MCID 15, AUSCAN pain (0-100) >8 or AUSCAN function (0-100) > 4	Not statistically significant, estimate not provided.	Multivariable analysis	(o)
	Courties 2017[30]	2.6	HIGH	Deterioration from baseline scores AUSCAN/FIHOA	OR (95% CI) 1.41 (0.39,5.09) p=0.59	Univariable analysis	(o)
Alcohol consumption	Courties 2017[30]	2.6	HIGH	Deterioration from baseline scores AUSCAN/FIHOA	OR (95% CI) 1.01 (0.64, 1.9)	Univariable analysis	(o)
	Neuprez 2021[38]	2	HIGH	AUSCAN (0-300) change >MCID 15, AUSCAN pain (0-100) >8 or AUSCAN function (0-100) > 4	Not statistically significant, estimate not provided.	Multivariable analysis	(o)
BMI	Courties 2017[30]	2.6	HIGH	Deterioration from baseline scores AUSCAN/FIHOA	OR (95% CI) 1.38 (0.79,2.39) p=0.25	Univariable analysis	(o)
	Dieppe 2000[34]	8	HIGH	patient reported outcomes (better, same, worse)	Descriptive data (chi squared and significance at 5% level) NS	Univariable analysis	(o)
	Vanhaverbeke 2020[46]	9.7	HIGH	AUSCAN function (0–90), change ≥ 7.2 or FIHOA (0–30), change ≥ 1.8.	OR (95% CI) 0.99 (0.93,1.05) p=0.8	Univariable analysis	(o)
	Ding 2011[36]	5	HIGH	Incident/persistent pain: ≥1 joint thumb, index or middle finger or separately ring or little finger, vs reference group of no pain at either time point radial or separately ulnar digits	PR (95% CI) 1.23 (0.86,1.76)	Multivariable: age, occupation, BMI, smoking, leisure hand activity, work status, radiographic joint OA	(o)

	Siviero 2020[37]	1.5	LOW	MCID score of >4 AUSCAN function	RR (95% CI) 0.95 (0.79,1.15) p=0.62	Multivariable: sex, age, country, education level	(o)
	Neuprez 2021[38]	2	HIGH	AUSCAN (0-300) change >MCID 15,<br>AUSCAN pain (0-100) >8 or<br>AUSCAN function (0-100) > 4	Not statistically significant, estimate not provided.	Multivariable analysis	(o)
Waist circumference	Neuprez 2021[38]	2	HIGH	AUSCAN (0-300) change >MCID 15,<br>AUSCAN pain (0-100) >8 or<br>AUSCAN function (0-100) > 4	Not statistically significant, estimate not provided.	Multivariable analysis	(o)
Osteoporosis	Siviero 2020[37]	1.5	LOW	MCID score of >4 AUSCAN function	RR (95% CI) 0.92 (0.75,1.14) p=0.45	Multivariable: sex, age, country, education level	(o)
Menopausal status	Courties 2017[30]	2.6	HIGH	Deterioration from baseline scores AUSCAN/FIHOA	OR (95% CI) 0.75 (0.4,1.44) p=0.39	Univariable analysis	(o)
	Neuprez 2021[38]	2	HIGH	AUSCAN (0-300) change >MCID 15,<br>AUSCAN pain (0-100) >8 or<br>AUSCAN function (0-100) > 4	Not statistically significant, estimate not provided.	Multivariable analysis	(o)
Use of analgesics /NSAIDs	Siviero 2020[37]	1.5	LOW	MCID score of >4 AUSCAN function	RR (95% CI) 1.01 (0.83,1.21) p=0.96	Multivariable: sex, age, country, education level	(o)
	Dieppe 2000[34]	8	HIGH	patient reported outcomes (better, same, worse)	Descriptive data (chi squared and significance at 5% level). Patients using NSAIDs were more likely to report improvement (24%) than those not using NSAIDs (12%) p=0.017	Univariable analysis	(-)
**Symptom severity**							
Baseline self-reported pain & function	Bijsterbosch 2011[39]	6	LOW	Poor outcome not fulfilling PASS for AUSCAN (> 8.2, 16.1)	RR (95% CI) ∞<br>Pain: pain>8: 5.74 (4.38,6.65)<br>function >16: 2.57 (1.26,4.13);<br>Function: pain >8: 3.56 (1.63,5.83),<br>function >16: 6.88 (5.3,7.9)	Multivariable: baseline scores of the clinical outcome measure, follow-up time, family effects	(+)
	Siviero 2020[37]	1.5	LOW	MCID score of >4 AUSCAN function	RR (95%CI) pain score ≥5 1.11 (0.89,1.37) p=0.35	Multivariable: sex, age, country, education level	(o)

	Vanhaverbeke 2020[46]	9.7	HIGH	AUSCAN function (0–90), change ≥ 7.2 or FIHOA (0–30), change ≥ 1.8.	OR (95% CI) VAS >33mm 1.0 (0.34,2.94) p=0.995<br>FIHOA 0.85 (0.77,0.94) p=0.002	Univariable analysis	(o) pain<br><br>(-) functio n
	Courties 2017[30]	2.6	HIGH	Deterioration from baseline scores AUSCAN/FIHOA	OR (95% CI) 0.956 (0.88,1.038) p=0.28	Multivariable: no adjustment, FIHOA, CHD & depression	(o)
	Neuprez 2021[38]	2	HIGH	AUSCAN (0-300) change >MCID 15,<br>AUSCAN pain (0-100) >8 or<br>AUSCAN function (0-100) > 4	OR (95% CI) AUCAN score <74.5 1.02 (1.01,1.03) P<0.01.<br>AUSCAN pain score <47 OR 1.03 (1.01, 1.04) P<0.01,<br>AUSCAN function score <56 OR 1.02 (0.01, 0.03) p<0.01	Multivariable analysis	(+)
**Clinical Signs (examination)**							
Number of painful joints	Bijsterbosch 2011[39]	6	LOW	Poor outcome not fulfilling PASS for AUSCAN (> 8.2, 16.1)	RR (95% CI) ∞<br>Poor outcome pain >8: 2.11 (1.25,3.08)<br>poor outcome function >8: 2.39 (1.47,3.37)	Multivariable: baseline scores of the clinical outcome measure, follow-up time, family effects	(+)
	Neuprez 2021[38]	2	HIGH	AUSCAN (0-300) change >MCID 15,<br>AUSCAN pain (0-100) >8 or<br>AUSCAN function (0-100) > 4	Not statistically significant, estimate not provided.	Multivariable analysis	(o)
Number of painful nodes (Heberdens & Bouchards)	Bijsterbosch 2011[39]	6	LOW	Poor outcome not fulfilling PASS for AUSCAN (> 8.2, 16.1)	RR (95% CI) ∞<br>Poor outcome for pain >11: 1.44 (0.91,2.02)<br>poor outcome function >11: 0.98 (0.56,1.57)	Multivariable: baseline scores of the clinical outcome measure, follow-up time, family effects	(o)
	Neuprez 2021[38]	2	HIGH	AUSCAN (0-300) change >MCID 15,<br>AUSCAN pain (0-100) >8 or<br>AUSCAN function (0-100) > 4	Not statistically significant, estimate not provided.	Multivariable analysis	(o)

Pain Intensity on palpation	Bijsterbosch 2011[39]	6	LOW	Poor outcome not fulfilling PASS for AUSCAN (> 8.2, 16.1)	RR (95% CI) ∞<br>poor outcome for pain >4: 1.24 (0.68,1.99)<br>poor outcome for function >4: 1.46 (0.77,2.37)	Multivariable: baseline scores of the clinical outcome measure, follow-up time, family effects	(o)
Presence of tenderness Y/N	Vanhaverbeke 2020[46]	9.7	HIGH	AUSCAN function (0–90), change ≥ 7.2 or FIHOA (0–30), change ≥ 1.8.	OR (95% CI) 1.26 (0.38, 4.18) p=0.704	Univariable analysis	(o)
Number of tender joints	Neuprez 2021[38]	2	HIGH	AUSCAN (0-300) change >MCID 15,<br>AUSCAN pain (0-100) >8 or<br>AUSCAN function (0-100) > 4	Not statistically significant, estimate not provided.	Multivariable analysis	(o)
Presence of soft tissue swelling	Vanhaverbeke 2020[46]	9.7	HIGH	AUSCAN function (0–90), change ≥ 7.2 or FIHOA (0–30), change ≥ 1.8.	OR (95% CI) 1.32 (0.51,3.43) p=0.57	Univariable analysis	(o)
Number of swollen hand joints	Neuprez [30]2021[38]	2	HIGH	AUSCAN (0-300) change >MCID 15,<br>AUSCAN pain (0-100) >8 or<br>AUSCAN function (0-100) > 4	Not statistically significant, estimate not provided.	Multivariable analysis	(o)
Grip strength	Vanhaverbeke 2020[46]	9.7	HIGH	AUSCAN function (0–90), change ≥ 7.2 or FIHOA (0–30), change ≥ 1.8.	OR (95% CI) 0.9 (0.9,1.06) p=0.586	Univariable analysis	(o)
	Siviero 2020[37]	1.5	LOW	MCID score of >4 AUSCAN function	RR (95% CI) Grip [ref >35kg]<br>< 20.5 kg 1.74: (1.17, 2.59) p=0.006<br>20.5-26.75kg: 1.78 (1.23,2.58 p=0.002<br>26.75-35kg: 1.61(1.17,2.24) p=0.04	Multivariable: sex, age, country, education level	(+)*
**Investigations**							
Imaging (radiograph/ MRI severity)	Bijsterbosch 2011[39]	6	LOW	Poor outcome not fulfilling PASS for AUSCAN (> 8.2, 16.1)	RR (95% CI) ∞<br>pain osteophytes >11: 1.22 (0.77,1.73)<br>JSN >22: 1.02 (0.64,1.46)<br>TBOA 0.92 (0.56,1.38)<br>function osteophytes >11: 0.72 (0.4,1.19) JSN>22: 0.79 (0.45,1.23)	Multivariable: baseline scores of the clinical outcome measure, follow-up time, family effects	(o)

	Courties 2017[30]	2.6	HIGH	Deterioration from baseline scores AUSCAN/FIHOA	OR (95% CI) function 0.99 (0.97,1.01) p=0.24	Univariable analysis	(o)
	Neuprez 2021[38]	2	HIGH	AUSCAN (0-300) change >MCID 15, AUSCAN pain (0-100) >8 or AUSCAN function (0-100) > 4	Not statistically significant, estimate not provided.	Multivariable analysis	(o)
	Van Beest 2021[49]	2	HIGH	increased thumb base pain on palpation (0-3)	(osteophytes) ORs (95%CI) 1.73(0.73, 4.1)	Multivariable analysis adjustments for other imaging scores.	(o)
Erosive OA (number of baseline E joints & R joints)	Vanhaverbeke 2020[46]	9.7	HIGH	AUSCAN function (0–90), change ≥ 7.2 or FIHOA (0–30), change ≥ 1.8.	OR (95% CI) Function total R joints 1.06 (0.85,1.32) p=0.617<br>total E joints 1.15 (0.86,1.54) p=0.339	Univariable analysis	(o)
	Neuprez 2021[38]	2	HIGH	AUSCAN (0-300) change >MCID 15, AUSCAN pain (0-100) >8 or AUSCAN function (0-100) > 4	OR (95% CI) Function total ≥4 R & E joints at baseline 2.26 (1.07, 4.78) p = 0.03	Multivariable analysis	(+)
Hand OA subset	Bijsterbosch 2011[39]	6	LOW	Poor outcome not fulfilling PASS for AUSCAN (> 8.2, 16.1)	RR (95% CI) TBOA 0.92 (0.56,1.38)<br>erosive 1.12 (0.5,1.73)<br>nodal 1.25 (0.82,1.74)	Multivariable: baseline scores of the clinical outcome measure, follow-up time, family effects	(o)
Presence of rheumatoid factor	Dieppe 2000[34]	8	HIGH	patient reported outcomes (better, same, worse)	Descriptive data (chi squared and significance at 5% level) NS	Univariable analysis	(o)
**Co-morbidities**							
Presence of knee OA	Dieppe 2000[34]	8	HIGH	patient reported outcomes (better, same, worse)	Descriptive data severe pain more common at all time points with knee & hand OA<br>8 years: 37% with severe pain (hand & knee group) vs. 27% knee alone 18% hand alone p<0.005	Univariable analysis	(+)
OA in other joints	Neuprez 2021[38]	2	HIGH	AUSCAN (0-300) change >MCID 15, AUSCAN pain (0-100) >8 or AUSCAN function (0-100) > 4	Not statistically significant, estimate not provided.	Multivariable analysis	(o)

Hypertension	Dieppe 2000[34]	8	HIGH	patient reported outcomes (better, same, worse)	Descriptive data (chi squared and significance at 5% level) NS	Univariable analysis	(o)
	Courties 2017[30]	2.6	HIGH	Deterioration from baseline scores AUSCAN/FIHOA	OR (95% CI) 1.1 (0.64,1.9) p=0.72	Univariable analysis	(o)
	Neuprez 2021[38]	2	HIGH	AUSCAN (0-300) change >MCID 15,<br>AUSCAN pain (0-100) >8 or<br>AUSCAN function (0-100) > 4	Not statistically significant, estimate not provided.	Multivariable analysis	(o)
Coronary Heart Disease	Courties 2017[30]	2.6	HIGH	Deterioration from baseline scores AUSCAN/FIHOA	OR (95% CI) 2.91 (1.02,8.26) p=0.045	Multivariable: not specified which adjustments were made.	(+)*
	Siviero 2020[37]	1.5	LOW	MCID score of >4 AUSCAN function	RR (95% CI) 1.05 (0.88,1.25) p=0.62	Multivariable: sex, age, country, education level	(o)
	Scherzer 2020[42]	12	HIGH	Transitions from AUSCAN pain categories <=6 "low" or AUSCAN >6 "high"<br>AUSCAN function sub score <=9 was defined as "better function"<br>AUSCAN function sub score >9 was defined as "worse function."	HR (95% CI) 0.99 (0.63,1.55)<br><br>HR (95% CI) 3.55 (0.88,14.3)	Multivariable: age, sex, race, education level, BMI, symptomatic KOA, NSAID use	(o)
	Neuprez 2021[38]	2	HIGH	AUSCAN (0-300) change >MCID 15,<br>AUSCAN pain (0-100) >8 or<br>AUSCAN function (0-100) > 4	Not statistically significant, estimate not provided.	Multivariable analysis	(o)
Single or multiple co-morbidities: DM, CVD or obesity	Scherzer 2020[42]	12	HIGH	Transitions in AUSCAN pain categories <=6 "low" or AUSCAN >6 "high"<br>AUSCAN function sub score <=9 was defined as "better function"<br>AUSCAN function sub score >9 was defined as "worse function."	HR (95% CI) for 1-2 co-morbidity: 4.05 (1.52,10.84)<br>HR (95% CI) for 2 co-morbidities: 3.98 (1.07,14.85)	Multivariable: age, sex, race, education level, BMI, symptomatic KOA, NSAID use	(+)
Cognitive impairment/ chronic lung disease/	Siviero 2020[37]	1.5	LOW	MCID score of >4 AUSCAN function	RR (95% CI) e.g. for lung disease 1 (0.88,1.25) p=0.98, all no association and NS individually	Multivariable: sex, age, country, education level	(o)

CVD/ PVD/ CVA/cancer							
Stroke/hypertension/ dyslipidaemia/ obesity	Courties 2017[30]	2.6	HIGH	Deterioration from baseline scores AUSCAN/FIHOA	OR (95%CI) e.g., stroke 0.91 (0.19,4.47) p=0.91, all no association and NS univariate analyses	Univariable analysis	(o)
dyslipidaemia	Neuprez 2021[38]	2	HIGH	AUSCAN (0-300) change >MCID 15,<br>AUSCAN pain (0-100) >8 or<br>AUSCAN function (0-100) > 4	Not statistically significant, estimate not provided.	Multivariable analysis	(o)
Stroke (CVA)	Neuprez 2021[38]	2	HIGH	AUSCAN (0-300) change >MCID 15,<br>AUSCAN pain (0-100) >8 or<br>AUSCAN function (0-100) > 4	Not statistically significant, estimate not provided.	Multivariable analysis	(o)
Diabetes	Courties 2017[30]	2.6	HIGH	Deterioration from baseline scores AUSCAN/FIHOA	OR (95%CI) 0.84 (0.35,2.01) p=0.69	Univariable analysis	(o)
	Scherzer 2020[42]	12	HIGH	Transitions in AUSCAN pain categories <=6 “low” or AUSCAN >6 “high”<br>AUSCAN function sub score <=9 was defined as “better function”<br>AUSCAN function sub score >9 was defined as “worse function.”	No. with diabetes/ no. without<br>HR (95% CI) worsening AUSCAN pain: 11/25 5.08 (1.38,18.77)	Multivariable: age, sex, race, education level, BMI, symptomatic KOA, NSAID use	(+)
	Siviero 2020[37]	1.5	LOW	MCID score of >4 AUSCAN function	Adj RR (95% CI) 0.86 (0.65,1.12) p=0.25	Multivariable: sex, age, country, education level	(o)
	Neuprez 2021[38]	2	HIGH	AUSCAN (0-300) change >MCID 15,<br>AUSCAN pain (0-100) >8 or<br>AUSCAN function (0-100) > 4	Not statistically significant, estimate not provided.	Multivariable analysis	(o)
**Psychological**							
Coping strategies (CORS)	Liu 2016[45]	1	HIGH	Disability present or absent, FIHOA >5	OR (95% CI) ∞<br>for disability using coping strategies:<br>pain decreasing activity>18: 5.68 (1.52,21.19)<br>limitations pacing >27: 5.0 (1.45,17.30)	Multivariable: sex, age, BMI, pain on palpation, limited motion and FIHOA baseline	(+)

					all other coping strategies no association.		
Anxiety	Siviero 2020[37]	1.5	LOW	MCID score of >4 AUSCAN function	RR (95%CI) 0.87(0.62,1.23) p=0.44	Multivariable: sex, age, country, education level	(o)
Depression	Siviero 2020[37]	1.5	LOW	MCID score of >4 AUSCAN function	RR (95%CI) 1.14 (0.90,1.44) p=0.29	Multivariable: sex, age, country, education level	(o)
	Courties 2017[30]	2.6	HIGH	Deterioration from baseline scores AUSCAN/FIHOA	OR (95% CI) 0.44 (0.18,1.05) p=0.06	Univariate analysis	(o)
	Neuprez 2021[38]	2	HIGH	AUSCAN (0-300) change >MCID 15, AUSCAN pain (0-100) >8 or AUSCAN function (0-100) > 4	Not statistically significant, estimate not provided.	Multivariable analysis	(o)
Illness Perception	Damman 2018[28]	2	LOW	AUSCAN function the MCII i.e. 1.4. For FIHOA, MCII or MCID is unknown, progression was defined as the minimal change potentially detectable, which is 1 unit.	RR (95% CI) ∞<br>IPQR identity subscale: 6-13 FIHOA 2.5 (1.1,5.7) timeline cyclical subscale: FIHOA 1.5 (1.0,2.4)<br>Other subscales and AUSCAN no positive associations on multivariable modelling with 3 factors adjusted for	Multivariable: age, Doyle Index, baseline AUSCAN function/FIHOA	(+)

Key: NS Statistically non-significant, MCII minimum clinically important improvement, PASS patient acceptable symptom state, OR odds ratio, RR risk ratio, PR prevalence ratio, HR hazard ratio, CI confidence interval, Adj adjusted analysis, unadj unadjusted analysis, NA not applicable, NS not significant, CORS coping with rheumatic stressors survey, NSAIDs non-steroidal anti-inflammatory drugs, FIHOA functional index for hand OA, AUSCAN Australian Canadian hand osteoarthritis index, BMI body mass index, KOA knee osteoarthritis, KLG Kellgren-Lawrence radiographic grading system, nos numbers of, BMLs bone marrow lesions, MCID minimally clinically important difference.

* multivariable analyses where a significant direction effect is report.

∞ indicates estimates for the highest tertile of each prognostic factor values are shown.

Supplementary Table 4. Amended GRADE assessment summary of findings for all prognostic factors with a relationship to an outcome (in at least one study).

**Potential Prognostic Factor identified (study references)**	**Number of participants**	**Number of studies**	**Phase of study***	**Association with hand OA prognosis**	**1 QUIPS ROB**	**2 Inconsistency**	**3 Imprecision**	**Strength of Evidence**
**Outcome – persistent or worsening pain**								
Self-reported hand Pain[38, 39]	560	2	1	(+) Adj RR 95% CI pain > 8: 5.74 (4.38,6.65)<br>(+) Unadj OR (95%CI) pain <47 1.03 (1.01, 1.04) P<0.01	-1	-1	+1	⊕■■low
Self-reported hand Function[39]	357	1	1	(+) Adj RR 95% CI function >16: 2.57 (1.26,4.13)	+1	-1	-1	⊕■■low
High hand use in leisure time (high/low)[36]	543	1	1	(+) Adj PR (95%CI) left hand:1.68 (1.00,2.83)	-1	-1	-1	■■■ very low
Number of painful hand joints[38, 39]	560	2	1	(+) Adj RR no. painful joints >8: 2.11 (1.25,3.08)<br>(o) estimate not provided	-1	-1	+1	⊕■■low
Presence of Diabetes[42 38]	1075	2	2	(+) Adj HR: 5.08 (1.38,18.77)<br>(o) estimate not provided	-1	+1	+1	⊕⊕■moderate
Presence of Knee OA[34]	500	1	1	(+) No model: Greater proportions with severe pain at all time points in knee & hand OA group (p<0.005)	-1	-1	-1	■■■ very low
**Outcome - poor function**								
Age at onset[46]	270	1	1	(-) Unadj OR (95%CI): 0.93 (0.87, 0.99) p=0.002 (older age at onset is protective)	-1	+1	-1	⊕■■low
Self-reported Hand Pain[37, 39, 46.]	3569	3	1	(+) Adj RR 95% CI: AUSCAN>8: 3.56 (1.63, 5.83)<br>(o) Adj RR AUSCAN ≥5 1.11 (0.89,1.37) p=0.35<br>(o) Unadj OR (95% CI) VAS >33mm: 1.00 (0.34,2.94) p= 0.995	+1	-1	+1	⊕⊕■moderate

Self-reported Hand Function[30, 38, 39, 46]	1699	4	1	(+) Adj RR (95%CI) >16: AUSCAN 6.88 (5.3-7.9)<br>(-) Unadj OR (95%CI): 0.85 (0.77, 0.94) p=0.002<br>(o) adj OR: 0.956 (0.88,1.038)<br>(o) estimate not provided	-1	-1	+1	⊕▪▪low
Number of painful hand joints[38, 39].	560	2	1	(+) Adj RR no. painful joints >8: 2.39 (1.47-3.37)<br>(o) estimate not provided	-1	-1	+1	⊕▪▪low
Number of erosive hand joints[38, 46].	473	2	1	(o) unadj OR (95% CI) total R joints 1.06 (0.85,1.32) p=0.617<br>total E joints 1.15 (0.86,1.54) p=0.339<br>(+) adj OR (95% CI) total ≥4 R & E joints 2.26 (1.07,4.78) p = 0.03	-1	-1	+1	⊕▪▪low
Grip Strength[37, 46]	3212	**2**	1	(+) Adj RR (95%CI) [ref >35kg] < 20.5 kg: 1.74 (1.17,2.59) p=0.006,<br>20.5-26.75kg: 1.78 (1.23, 2.58) p=0.002,<br>26.75-35kg: 1.61 (1.17-2.24) p=0.004<br>(o) Unadj OR (95% CI): 0.9 (0.9, 1.06) p=0.586	-1	-1	+1	⊕▪▪low
Presence of Coronary Heart Disease[30, 37, 38, 42]	4886	4	1	(o) Adj HR (95% CI): 3.55 (0.88,14.3)<br>(o) Adj RR (95% CI): 1.05 (0.88,1.25) p=0.62<br>(+) Adj OR (95%CI): 2.91 (1.02,8.26) p=0.045<br>(o) estimate not provided	-1	-1	+1	⊕▪▪low
Number of comorbidities[42]<br><br>(diabetes, cardiovascular disease, obesity)	872	1	2	Presence of 1 comorbidity (+) Adj HR (95%CI): 4.05 (1.52,10.84)	-1	-1	-1	▪▪▪<br>very low

				Presence of 2 comorbidities (+) Adj HR (95%CI): 3.98(1.07, 14.85)<br>Presence of 3 comorbidities (o) estimate not provided				
Income[37]	2942	1	1	(-) RR (95% CI) 0.6(0.36, 0.99) p=0.05	+1	-1	-1	⊕■■low
Coping Strategies (CORS)[45]	314	1	2	(+) Adj OR (95%CI) pain decreasing activity >18: 5.68(1.52,21.19)<br>Limitations pacing > 27: 5 (1.45,17.30)	-1	-1	-1	■■■<br>very low
Illness Perception[28]	384	1	2	(+) Adj RR (95% CI)<br>Identity 6-13: 2.5(1.1, 5.7)<br>Timeline: 1.5 (1.0, 2.4)	+1	-1	-1	⊕■■low
**Outcome: global change in condition better, same, or worse**								
Disease duration[34]	500	1	1	(+) No model: 5 years longer disease duration in those reporting worse outcome (p=0.007)	-1	-1	-1	■■■ very low
Use of NSAIDs[34]	500	1	1	(-) patient using NSAIDs were more likely to report improvement (24%) vs 12% p=0.017	-1	-1	-1	■■■ very low
**Outcome: Additional Symptomatic Hand Joint**								
Ethnicity[48]	327	1	2	(+) White vs African American adj OR 1.96 (1.01, 3.83)	-1	+1	-1	⊕■■low

Key: *Phase of study: 1= exploratory prognostic study, 2 = confirmatory prognostic study

OR odds ratio, RR risk ratio, HR hazard ratio, PR prevalence ratio, CI confidence interval, Adj adjusted analysis, unadj unadjusted analysis, CORS coping with rheumatic stressors survey, NSAIDs non-steroidal anti-inflammatory drugs, AUSCAN Australian Canadian hand osteoarthritis index, VAS Visual analogue scale, MRI Magnetic resonance imaging, E erosive, R Remodelled.

*Phase of study: 1= exploratory prognostic study, 2 = confirmatory prognostic study

Conceptualization: Quality of evidence across studies

⊕⊕⊕ High = Further research is very unlikely to change our confidence in the estimate of effect.

⊕⊕■ Moderate = Further research is likely to have an important impact on our confidence in the estimate of effect and may change the estimate.

⊕■■ Low = Further research is very likely to have an important impact on our confidence in the estimate of effect and is likely to change the estimate.

■■■ Very low = Any estimate of effect is very uncertain
